# Biomarker vs MRI-Enhanced Strategies for Prostate Cancer Screening: The STHLM3-MRI Randomized Clinical Trial

**DOI:** 10.1001/jamanetworkopen.2024.7131

**Published:** 2024-04-22

**Authors:** Lars Björnebo, Andrea Discacciati, Ugo Falagario, Hari T. Vigneswaran, Fredrik Jäderling, Henrik Grönberg, Martin Eklund, Tobias Nordström, Anna Lantz

**Affiliations:** 1 Department of Medical Epidemiology and Biostatistics, Karolinska Institutet, Stockholm, Sweden; 2 Department of Molecular Medicine and Surgery, Karolinska Institutet, Stockholm, Sweden; 3 Department of Urology and Kidney Transplantation, University of Foggia, Foggia, Italy; 4 Department of Diagnostic Radiology, Karolinska Institutet, Stockholm, Sweden; 5 Department of Clinical Sciences, Danderyd Hospital, Danderyd, Sweden

## Abstract

**Importance:**

Prostate cancer guidelines often recommend obtaining magnetic resonance imaging (MRI) before a biopsy, yet MRI access is limited. To date, no randomized clinical trial has compared the use of novel biomarkers for risk estimation vs MRI-based diagnostic approaches for prostate cancer screening.

**Objective:**

To evaluate biomarker-based risk estimation (Stockholm3 risk scores or prostate-specific antigen [PSA] levels) with systematic biopsies vs an MRI-enhanced strategy (PSA levels and MRI with systematic and targeted biopsy) for the detection of clinically significant prostate cancer in a screening setting.

**Design, Setting, and Participants:**

This open-label randomized clinical trial conducted in Stockholm, Sweden, between April 4, 2018, and December 10, 2020, recruited men aged 50 to 74 years with no history of prostate cancer. Participants underwent blood sampling for PSA and Stockholm3 tests to estimate their risk of clinically significant prostate cancer (Gleason score ≥3 + 4). After the blood tests were performed, participants were randomly assigned in a 2:3 ratio to receive a Stockholm3 test with systematic biopsy (biomarker group) or a PSA test followed by MRI with systematic and targeted biopsy (MRI-enhanced group). Data were analyzed from September 1 to November 5, 2023.

**Interventions:**

In the biomarker group, men with a Stockholm3 risk score of 0.15 or higher underwent systematic biopsies. In the MRI-enhanced group, men with a PSA level of 3 ng/mL or higher had an MRI and those with a Prostate Imaging–Reporting and Data System (PI-RADS) score of 3 or higher (range: 1-5, with higher scores indicating a higher likelihood of clinically significant prostate cancer) underwent targeted and systematic biopsies.

**Main Outcomes and Measures:**

Primary outcome was detection of clinically significant prostate cancer (Gleason score ≥3 + 4). Secondary outcomes included detection of clinically insignificant cancer (Gleason score ≤6) and the number of biopsy procedures performed.

**Results:**

Of 12 743 male participants (median [IQR] age, 61 [55-67] years), 5134 were assigned to the biomarker group and 7609 to the MRI-enhanced group. In the biomarker group, 8.0% of men (413) had Stockholm3 risk scores of 0.15 or higher and were referred for systematic biopsies. In the MRI-enhanced group, 12.2% of men (929) had a PSA level of 3 ng/mL or higher and were referred for MRI with biopsies if they had a PI-RADS score of 3 or higher. Detection rates of clinically significant prostate cancer were comparable between the 2 groups: 2.3% in the biomarker group and 2.5% in the MRI-enhanced group (relative proportion, 0.92; 95% CI, 0.73-1.15). More biopsies were performed in the biomarker group than in the MRI-enhanced group (326 of 5134 [6.3%] vs 338 of 7609 [4.4%]; relative proportion, 1.43 [95% CI, 1.23-1.66]), and more indolent prostate cancers were detected (61 [1.2%] vs 41 [0.5%]; relative proportion, 2.21 [95% CI, 1.49-3.27]).

**Conclusions and Relevance:**

Findings of this randomized clinical trial indicate that combining a Stockholm3 test with systematic biopsies is comparable with MRI-based screening with PSA levels and systematic and targeted biopsies for detection of clinically significant prostate cancer, but this approach resulted in more biopsies as well as detection of a greater number of indolent cancers. In regions where access to MRI is lacking, the Stockholm3 test can aid in selecting patients for systematic prostate biopsy.

**Trial Registration:**

ClinicalTrials.gov Identifier: NCT03377881

## Key Points

**Question:**

Is use of a blood-based biomarker with systematic biopsies a viable alternative to using prostate-specific antigen (PSA) levels and magnetic resonance imaging (MRI) to screen for prostate cancer?

**Findings:**

In this randomized clinical trial of 12 743 men with no previous prostate cancer diagnosis, a blood-based biomarker (Stockholm3 risk score ≥0.15) combined with systematic biopsies detected clinically significant prostate cancer at levels comparable with MRI-targeted biopsies based on PSA levels. However, the biomarker-based approach resulted in more biopsies and detected a greater number of indolent cancers.

**Meaning:**

Findings of this study suggest that in regions with limited access to MRI, use of blood-based biomarkers could be a feasible alternative for prostate cancer screening.

## Teaser

This randomized clinical trial compared use of a biomarker-based strategy vs a magnetic resonance imaging–enhanced strategy for detection of clinically significant prostate cancer among men in Sweden.

## Introduction

Population-based screening with a prostate-specific antigen (PSA) test has been shown to reduce mortality in prostate cancer.^1^ However, the PSA test has limited sensitivity and specificity, resulting in missed early detection of significant cancers, unnecessary biopsies, and detection of indolent cancers. There is level-1 evidence from multiple studies, including the STHLM3-MRI (Prostate Cancer Screening With PSA, the Stockholm3 Test, and MRI) trial on which the present trial is based, that performing magnetic resonance imaging (MRI) in patients with moderately elevated PSA levels can reduce unnecessary biopsies while maintaining the ability to detect clinically significant prostate cancer.^2,3,4,5^ Current guidelines from the European Association of Urology^6^ and the National Comprehensive Cancer Network^7^ recommend obtaining an MRI of the prostate before diagnostic biopsy. The American Urological Association^8^ considers MRI optional before initial prostate biopsy but recommends an MRI for men with a previous negative biopsy result.

Nevertheless, MRI-based screening for prostate cancer presents challenges. The procedure is technically complex, patient access may vary, and most studies have been conducted at specialized centers with extensive experience in MRI of the prostate, which raises concerns about the generalizability of findings across diverse settings. Additionally, radiologists’ assessments have exhibited substantial interobserver variability.^9,10,11^ Furthermore, there is ongoing debate about whether the pathological evaluation of targeted biopsy specimens should differ from that of systematic biopsy specimens given the potential for Gleason score inflation due to increased detection with targeted biopsy, with unclear long-term clinical implications.^12,13^

Over the past decade, several blood- and urine-based tests have emerged to refine patient selection for prostate biopsy, with many tests showing better sensitivity and specificity than PSA testing.^14,15,16,17,18^ These tests incorporate parameters such as genetic markers, PSA-related markers, and clinical variables. One such test, the blood-based Stockholm3 test, combines patient age, previous prostate biopsy results, family history of prostate cancer, single-nucleotide variations (formerly single-nucleotide polymorphisms), and levels of total PSA, free PSA, human kallikrein 2, β-microseminoprotein, and growth differentiation factor 15 to estimate the risk of clinically significant cancer (Gleason score ≥3 + 4). The Stockholm3 test was designed to detect clinically significant prostate cancer as well as PSA levels of 3 ng/mL or higher (to convert to micrograms per liter, multiply by 1), and a prospective population-based study found that the test could achieve this goal while reducing the number of biopsies performed and detection of clinically insignificant cancers.^16^ Additionally, Nordström et al^19^ showed within the MRI-enhanced group of the STHLM3-MRI trial that using the Stockholm3 and PSA tests in combination to determine which men should undergo MRI substantially reduced the number of MRIs and biopsies performed without compromising the detection of clinically significant prostate cancer.

As interest in prostate cancer screening grows and the limitations of PSA- and MRI-based screening become apparent, there is a need for alternative screening methods. The European Commission recently called on its members to establish organized prostate cancer screening programs. This randomized clinical trial represents an important step in this direction by assessing the effectiveness of blood-based risk estimation along with systematic biopsies and comparing this biomarker-based approach with the established MRI-targeted biopsy approach within a population-based screening context.

## Methods

### Study Design

The STHLM3-MRI trial was a population-based randomized clinical trial that used both paired and randomized designs to facilitate comparisons of multiple strategies for diagnosing prostate cancer. The study was conducted in Stockholm, Sweden, from April 4, 2018, to December 10, 2020. Detailed study methods have been published.^20^ Men aged 50 to 74 years who were residents of Stockholm County, Sweden, were eligible for inclusion. Exclusion criteria included a prior prostate cancer diagnosis, a prostate biopsy within 60 days before the study invitation, contraindications to MRI, and severe illness. The trial was approved by the Swedish Ethical Review Authority in Stockholm County and was monitored by an independent data safety and monitoring committee (Supplement 1). Written informed consent was obtained from all participants during the initial blood sample collection. We adhered to the Consolidated Standards of Reporting Trials (CONSORT) reporting guideline.

### Participant Recruitment and Baseline Assessment

Participants were randomly selected by Statistics Sweden and invited via mail to participate in the study. Between February 5, 2018, and March 4, 2020, 49 118 men received invitations, of whom 12 750 men expressed interest and met the eligibility criteria for the initial screening. The trial was conducted between April 4, 2018, and December 10, 2020. Baseline characteristics were collected through a secure web portal, where digital laboratory referrals were also created. Blood samples for measuring PSA levels and (if required) determining the Stockholm3 risk score were collected from all participants.

### Randomization

After baseline assessment, men with an elevated risk of prostate cancer (PSA level ≥3 ng/mL or Stockholm3 risk score ≥0.11) were randomly assigned in a 2:3 ratio to either the biomarker group or the MRI-enhanced group. Initally, men in the biomarker group with a Stockholm3 score of 0.11 or higher were referred for systematic biopsies. In the MRI-enhanced group, men whose PSA level was 3 ng/mL or higher were referred for MRI with targeted or systematic biopsies if they had a Prostate Imaging–Reporting and Data System (PI-RADS) score of 3 or higher (considered as positive MRI results). The PI-RADS is used to assess the likelihood of clinically significant prostate cancer for each lesion on a scale of 1 (very low) to 5 (very high).

**Figure 1.**
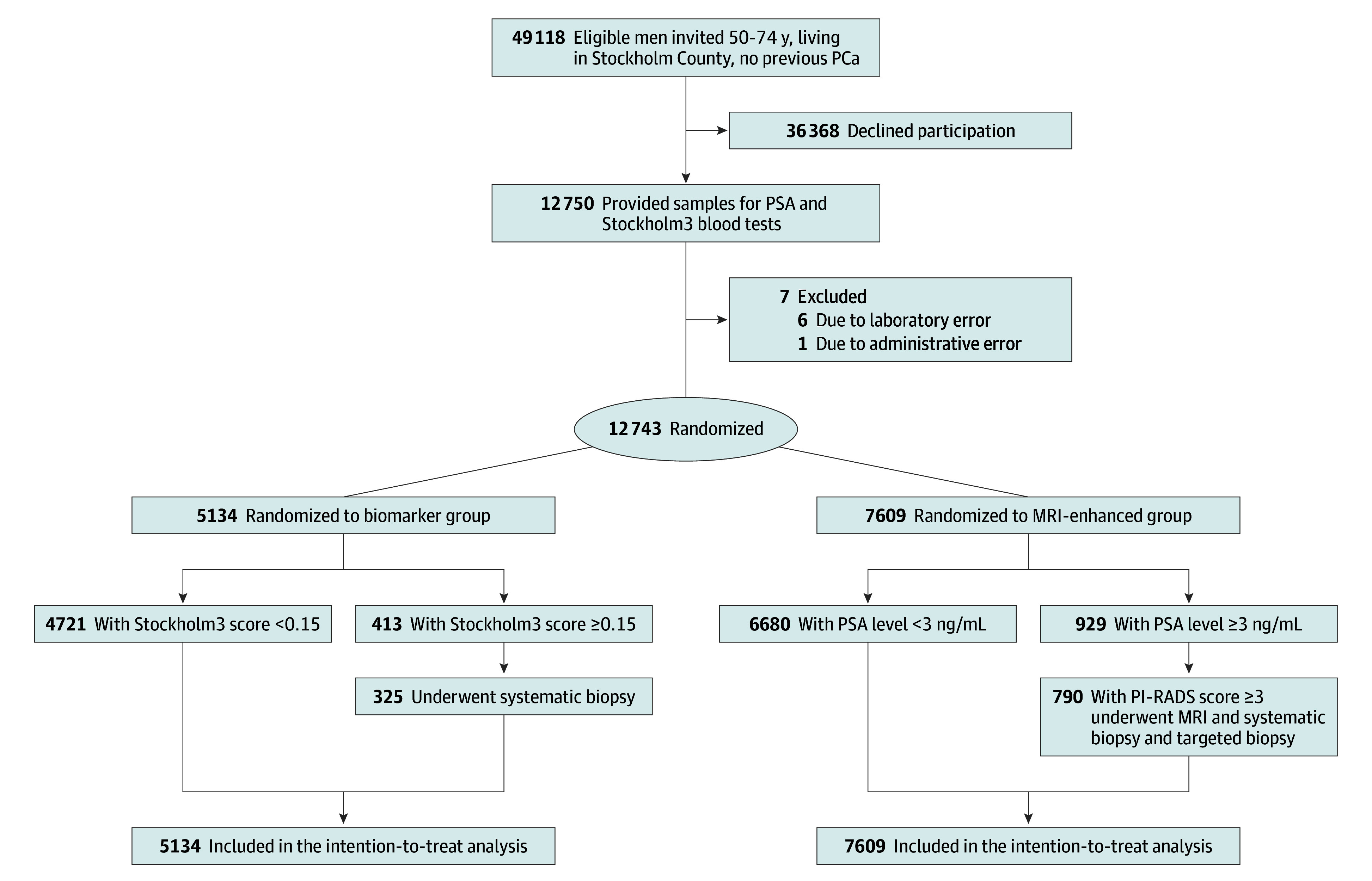
Trial Flowchart MRI indicates magnetic resonance imaging; PCa, prostate cancer; PI-RADS, Prostate Imaging–Reporting and Data System; and PSA, prostate-specific antigen. A PI-RADS score of 3 or higher was considered to be a positive MRI result (range: 1-5, with higher scores indicating a higher likelihood of clinically significant prostate cancer). To convert to micrograms per liter, multiply by 1.

Randomization lists were generated in blocked sequences that were stratified by sextiles for the risk of significant prostate cancer based on Stockholm3 risk score, with a block size of 5. The study biostatistician (A.D.) prepared the randomization lists, and participants were assigned to study groups by the database administrator. Subsequently, men without elevated PSA levels or Stockholm3 risk scores were also randomly assigned to the biomarker or MRI-enhanced groups (Figure 1). Participants and treating clinicians were not blinded to group assignment.

### Intervention

To analyze PSA levels and Stockholm3 risk scores, 12 mL of blood was collected in EDTA-containing tubes at 1 of 60 laboratories in Stockholm and sent to a central laboratory (A3P Biomedical, Uppsala, Sweden) for analysis. The PSA samples were analyzed using the BRAHMS Kryptor Compact Plus analyzer (Thermo Fisher Scientific). The Stockholm3 risk algorithm did not include measurement of prostate volume or performance of a digital rectal examination.

Experienced urologists performed systematic prostate biopsies using transrectal ultrasonography, obtaining 10 to 12 biopsy cores from the peripheral zone of the prostate (apical, midgland, and base). Men randomly assigned to the MRI-enhanced group underwent a T2- and diffusion-weighted biparametric MRI protocol involving a 1.5T or 3T scanner without an endorectal coil. Each case was reviewed by at least 2 experienced uroradiologists using PI-RADS, version 2.1 to assess areas suspicious for prostate cancer. An external uroradiologist reviewed 8% (99) of the MRI results. Men with a positive MRI result were referred for MRI-targeted biopsy assisted by dedicated software. No more than 3 areas suspicious for prostate cancer were chosen for targeted transrectal biopsy, with 3 to 4 biopsy cores obtained per area. Additionally, the same urologist performed transrectal systematic biopsies, obtaining 10 to 12 biopsy cores after the targeted procedure. Men with a negative MRI result (ie, PI-RADS score ≤2) did not undergo biopsy unless the Stockholm3 risk score was 0.25 or higher, indicating a high risk of clinically significant cancer. Prostatic tissue biopsy samples were reviewed by an experienced uropathologist, who reported the Gleason score and the extent of cancer (in millimeters) for each biopsy core.

### Outcomes

The primary outcome was the detection of clinically significant prostate cancer, defined as a Gleason score of 3 + 4 or higher. Secondary outcomes included the number of biopsies performed, the number of benign biopsies, clinically insignificant cancers detected (Gleason score ≤6), higher-grade cancers detected (Gleason score ≥4 + 3), and serious adverse events (ie, postbiopsy hospitalization due to infection). Participants were followed up for at least 200 days after PSA test results were reviewed, with additional monitoring for postbiopsy hospitalizations and postprostatectomy pathology.

### Statistical Analysis

The number of invited participants in the study was based on power calculations for demonstrating noninferiority in detecting clinically significant cancer in men with a PSA level of 3 ng/mL or higher across the study groups, as reported by Eklund et al.^5^ The STHLM3-MRI trial incorporated a paired step and a randomized design, allowing for the comparison of multiple diagnostic strategies. The analysis followed the statistical analysis plan in which the comparison of the diagnostic strategies was prespecified and approved by a data safety and monitoring committee.

The Stockholm3 risk score cutoff of 0.15, suggested by Nordström et al^19^ for sensitivity equivalent to that of a PSA level of 3 ng/mL or higher and MRI, was used in this study. Results with the original Stockholm3 cutoff of 0.11 and a PSA level of 3 ng/mL or higher (which is a standard cutoff value for evaluating biopsy specimens) were also included for comparison. For all outcomes, the proportions of men with abnormal blood test results (Stockholm3 risk score ≥0.15 or higher, Stockholm3 risk score ≥0.11, or PSA level ≥3 ng/mL, as appropriate) and with outcomes of interest in the biomarker group and the MRI-enhanced group were calculated. Relative proportions between proportions were assessed by computing their ratio and are reported as the proportion of men in the biomarker group relative to the MRI-enhanced group. The 95% CIs were calculated by exponentiating the normal-based confidence limits computed on the log scale. For a practical perspective on screening, the numbers of diagnostic tests, biopsy procedures, and diagnosed cases of prostate cancer (low-grade, clinically significant, and high-grade) per 10 000 screened men were calculated by multiplying the proportions derived from the initial analysis by 10 000.

The main analyses were performed in the intention-to-treat population, which included all enrolled and randomly assigned participants. A sensitivity analysis was conducted within a model-based multiple imputation population, where the primary outcome was imputed when missing. Another sensitivity analysis ignored biopsy outcomes for men in the MRI-enhanced group with negative MRI findings but who underwent biopsy due to a Stockholm3 risk score of 0.25 or higher. To account for potential Gleason score inflation, an alternative model exclusively used outcomes from systematic biopsies for men in the MRI-enhanced group who had undergone both systematic and targeted biopsies. All analyses were conducted using R, version 4.2.3 (R Project for Statistical Computing). Statistical significance was set at 2-sided *P* < .05. Data were analyzed from September 1 to November 5, 2023.

## Results

Of 12 750 male participants who provided blood samples for analysis, 12 743 (median [IQR] age, 61 [55-67] years) were randomly assigned to the biomarker group (5134) or to the MRI-enhanced group (7609). In the biomarker group, 8.0% (413 men) had an elevated Stockholm3 risk score (≥0.15) and were assigned to undergo systematic biopsies only. In the MRI-enhanced group, 12.2% (929 men) had a PSA level of 3 ng/mL or higher and were assigned to undergo MRI and biopsy if they had positive MRI results (Figure 1). Participants had a median (IQR) PSA level of 1.03 (0.60-1.88) ng/mL and a median (IQR) Stockholm3 risk score of 0.09 (0.06-0.14). The number of participants with a family history of prostate cancer, a previous prostate biopsy, or use of a 5-α reductase inhibitor did not differ significantly between the 2 groups (Table 1). Additionally, 34 men in the MRI-enhanced group with elevated PSA levels and negative MRI findings underwent systematic biopsies due to having a Stockholm3 risk score of 0.25 or higher.

**Table 1.** Patient Characteristics

Characteristic	Enrolled participants (N = 12 750)	Randomly assigned participants^a^	
		MRI-enhanced group (n = 7609)	Biomarker-based group (n = 5134)
Age, median (IQR), y	61 (55-67)	61 (55-67)	61 (55-67)
Prostate cancer in a first-degree relative, No. (%)			
Yes	1947 (17)	1140 (17)	806 (18)
No	9231 (83)	5528 (83)	3698 (82)
Previous prostate biopsy, No. (%)			
Yes	533 (4)	330 (5)	203 (4)
No	11 736 (96)	6998 (95)	4731 (96)
5-α Reductase inhibitor treatment, No. (%)			
Yes	255 (2.0)	172 (2.3)	83 (1.6)
No	12 344 (98)	7343 (98)	4994 (98)
PSA, median (IQR), ng/mL	1.03 (0.60-1.88)	1.03 (0.60-1.88)	1.02 (0.60-1.88)
Unknown	45	28	17
Stockholm3 risk score, median (IQR)	0.09 (0.06-0.14)	0.09 (0.06-0.14)	0.09 (0.06-0.14)
Unknown	8443	5037	3406
Prostate volume, median (IQR), mL	40 (30-55)	40 (31-55)	39 (30-52)
Unknown	10 821	6350	4464
PSA density, median (IQR), ng/mL^2^	0.08 (0.06-0.12)	0.08 (0.06-0.12)	0.09 (0.06-0.12)
Unknown	10 821	6350	4464
PI-RADS score, No. (%)			
1-2	815 (65)	815 (65)	0 (0)
3	261 (21)	261 (21)	0 (0)
4	115 (9.1)	115 (9.1)	0 (0)
5	70 (5.6)	69 (5.5)	1 (100)

Abbreviations: MRI, magnetic resonance imaging; PI-RADS, Prostate Imaging–Reporting and Data System (range: 1-5, with higher scores indicating higher likelihood of clinically significant prostate cancer); PSA, prostate-specific antigen.

SI conversion: To convert PSA to micrograms per liter, multiply by 1.

^a^ Seven men were not randomly assigned due to laboratory or administrative errors.

**Figure 2.**
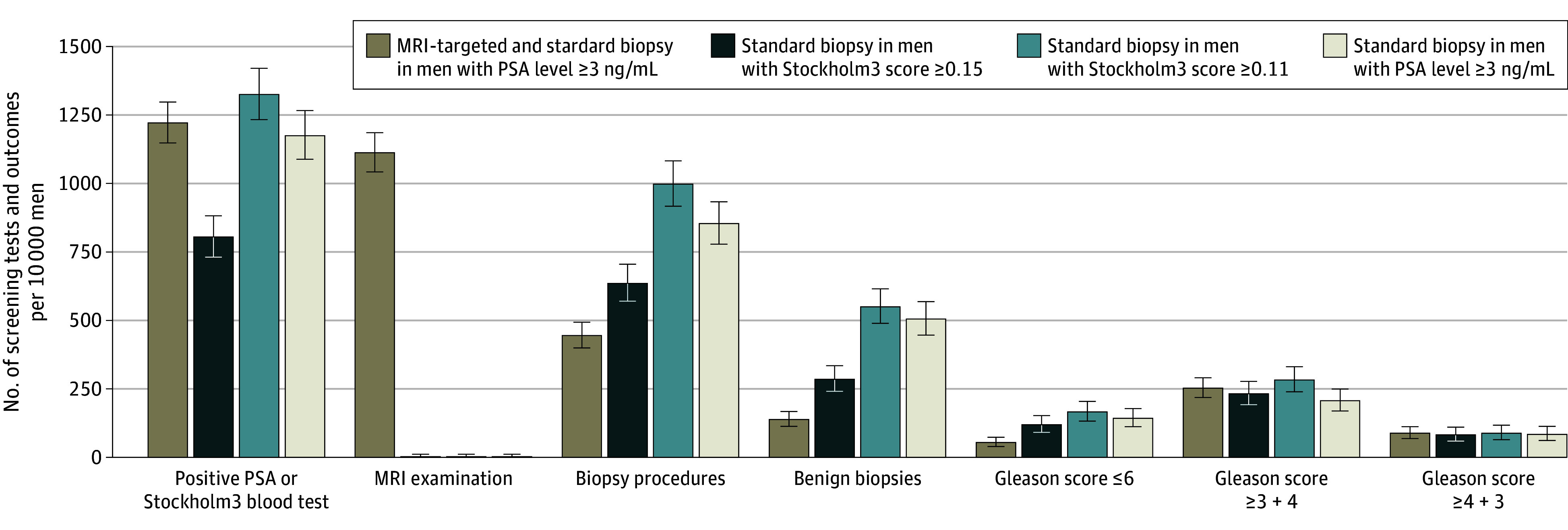
Screening Tests and Prostate Cancer Outcomes for the Different Diagnostic Strategies per 10 000 Screened Men Stockholm3 is a blood-based test for estimating the risk of clinically significant cancer. MRI indicates magnetic resonance imaging; PSA, prostate-specific antigen. Error bars indicate the 95% CIs. To convert PSA to micrograms per liter, multiply by 1.

Clinically significant prostate cancer was detected in 2.3% of men (119 of 5134) in the biomarker group (Stockholm3 risk score ≥0.15%) and in 2.5% of men (192 of 7609) in the MRI-enhanced group (PSA level ≥3 ng/mL), for a relative proportion of 0.92 (95% CI, 0.73-1.15). More men in the biomarker group underwent biopsy procedures than in the MRI-enhanced group (326 of 5134 [6.3%] vs 338 of 7609 [4.4%]; relative proportion, 1.43 [95% CI, 1.23-1.66]), and those in the biomarker group were more likely to have insignificant prostate cancers detected (61 [1.2%] vs 41 [0.5%]; relative proportion, 2.21 [95% CI, 1.49-3.27]). The mean (SD) number of biopsy cores did not differ significantly between the 2 groups (0.8 [2.9] biopsy cores in the biomarker group vs 0.7 [3.2] in the MRI-enhanced group, *P* = .12). The median (IQR) number of core specimens obtained per biopsy was 12 (12-12) in the biomarker-based group and 15 (15-16) in the MRI-enhanced group. Moreover, rates of postbiopsy hospitalization were similar between the 2 groups (0.2% in each group; relative proportion, 1.14 [95% CI, 0.50-2.60]). For men who underwent biopsy, the risk of postbiopsy hospitalization was 3.1% in the biomarker-based group and 3.8% in the MRI-enhanced group. Additionally, a higher proportion of men in the biomarker group (1.7%) than in the MRI-enhanced group (0.4%) who were referred for biopsy did not undergo the procedure (relative proportion, 4.61 [95% CI, 3.01-7.04]) (Table 2). Per 10 000 screened men, use of a Stockholm3 risk score of 0.15 or higher followed by systematic biopsies would reduce the number of MRI scans by 1100, but 191 additional biopsies would be required to find 65 additional low-grade prostate cancers while 20 clinically significant prostate cancers would be missed compared with the number missed with the use of PSA-level screening followed by MRI and biopsies (Figure 2).

**Table 2.** Outcomes of Patients With MRI Examinations, Biopsies, and Prostate Cancer According to Screening Strategies

Outcome	MRI-enhanced group (n = 7609), PSA ≥3 ng/mL, No. (%)	Biomarker-based group (n = 5134)					
		Stockholm3 risk score ≥0.15, No. (%)	Relative proportion (95% CI)	Stockholm3 risk score ≥0.11, No. (%)	Relative proportion (95% CI)	PSA ≥3 ng/mL, No. (%)	Relative proportion (95% CI)
Participants with elevated test results	929 (12.2)	413 (8.0)	0.66 (0.59 to 0.74)	680 (13.2)	1.08 (0.99 to 1.19)	603 (11.7)	0.96 (0.87 to 1.06)
MRI examinations	846 (11.1)	1 (<0.1)	NA	1 (<0.1)	NA	1 (<0.1)	NA
Biopsy procedures	338 (4.4)	326 (6.3)	1.43 (1.23 to 1.66)	512 (10.0)	2.25 (1.97 to 2.56)	438 (8.5)	1.92 (1.67 to 2.20)
Benign biopsy results	105 (1.4)	146 (2.8)	2.06 (1.61 to 2.64)	282 (5.5)	3.98 (3.19 to 4.97)	259 (5.0)	3.66 (2.92 to 4.57)
Gleason score 6 cancers	41 (0.5)	61 (1.2)	2.21 (1.49 to 3.27)	85 (1.7)	3.07 (2.12 to 4.45)	73 (1.4)	2.64 (1.80 to 3.86)
Gleason score ≥3 + 4 cancers (ITT)	192 (2.5)	119 (2.3)	0.92 (0.73 to 1.15)	145 (2.8)	1.12 (0.90 to 1.38)	106 (2.1)	0.82 (0.65 to 1.03)
Gleason score ≥3 + 4 cancers (MBI)	230 (3.0)	139 (2.7)	0.90 (0.68 to 1.11)	175 (3.4)	1.13 (0.92 to 1.33)	130 (2.5)	0.83 (0.61 to 1.06)
Gleason score ≥4 + 3 cancers	67 (0.9)	42 (0.8)	0.93 (0.63 to 1.36)	45 (0.9)	1.00 (0.68 to 1.45)	43 (0.8)	0.95 (0.65 to 1.39)
Referred for biopsy but no biopsy performed	28 (0.4)	87 (1.7)	4.61 (3.01 to 7.04)	168 (3.3)	8.89 (5.97 to 13.25)	165 (3.2)	8.73 (5.86 to 13.02)
No. of biopsy cores, mean (SD)	0.7 (3.2)	0.8 (2.9)	0.08 (−0.02 to 0.19)^a^	1.2 (3.6)	0.52 (0.40 to 0.64)^a^	1.0 (3.3)	0.34 (0.23 to 0.46)^a^
Postbiopsy hospitalizations	13 (0.2)	10 (0.2)	1.14 (0.50 to 2.60)	22 (0.4)	2.51 (1.26 to 4.97)	17 (0.3)	1.94 (0.94 to 3.99)

Abbreviations: ITT, intention-to-treat analysis; MBI; model-based imputation; MRI, magnetic resonance imaging; NA, not applicable; PSA, prostate-specific antigen.

^a^ Welch 2-sample *t* test.

Using a Stockholm3 risk score cutoff of 0.11 or higher, a greater number of clinically significant prostate cancers was detected compared with the MRI-enhanced approach, although this difference did not reach statistical significance (relative proportion, 1.12; 95% CI, 0.90-1.38). Furthermore, more biopsy procedures were required and a greater number of insignificant cancers was detected with the biomarker-based approach (Table 2). In the case of using PSA level in combination with systematic biopsies, this approach necessitated more biopsies but resulted in a lower detection rate of clinically significant prostate cancer compared with either the Stockholm3 risk score or the MRI-enhanced approach (Table 2). Importantly, none of the strategies in the biomarker-based group detected fewer clinically significant cancers (Gleason score ≥3 + 4) or higher-grade cancers (Gleason score ≥4 + 3) compared with the MRI-enhanced approach.

Detection of clinically significant prostate cancer for the model-based imputation sensitivity analysis did not differ from findings in the intention-to-treat analysis (Table 2). Excluding biopsy outcomes for men in the MRI-enhanced group with negative MRI results but who underwent biopsy because of a Stockholm3 risk score of 0.25 or higher did not alter the results (eTable 1 in Supplement 2). In the MRI-enhanced group, after limiting the analysis to only include outcomes from systematic biopsies in men with positive MRI results, clinically significant prostate cancer was detected in 1.9% of men in the MRI-enhanced group and 2.3% of those in the biomarker group (relative proportion, 1.20; 95% CI, 0.94-1.52) (eTables 2 and 4 and the eFigure in Supplement 2).

For men diagnosed with prostate cancer who underwent radical prostatectomy, the proportions of Gleason scores misclassified between biopsy review and final pathology examination were similar between the groups (40% in the biomarker group vs 38% in the MRI-enhanced group). While not statistically significant, tumor upgrading was more common in the biomarker group compared with the MRI-enhanced group (33% vs 23%, *P* = .17) and downgrading was less common (7% vs 14%, *P* = .15) (Table 3 and eTable 3 in Supplement 2).

**Table 3.** Reclassification of Gleason Score Between Initial Biopsy and Final Pathology Report for Men Undergoing Radical Prostatectomy

Outcome	MRI-enhanced group, PSA ≥3 ng/mL, No. (%) (n = 125)	Biomarker-based group					
		Stockholm3 risk score ≥0.15, No. (%) (n = 58)	*P* value^a^	Stockholm3 risk score ≥0.11, No. (%) (n = 70)	*P* value^a^	PSA ≥3 ng/mL, No. (%) (n = 58)	*P* value^a^
Reclassified	47 (38)	23 (40)	.79	28 (40)	.74	24 (41)	.63
Upgraded	29 (23)	19 (33)	.17	24 (34)	.10	19 (33)	.17
Downgraded	18 (14)	4 (7)	.15	4 (6)	.07	5 (9)	.27

Abbreviations: MRI, magnetic resonance imaging; PSA, prostate-specific antigen.

SI conversion: To convert PSA to micrograms per liter, multiply by 1.

^a^ Pearson χ^2^ test.

As previously reported by Nordström et al,^19^ the area under the curve for detecting clinically significant prostate cancer was 0.76 (95% CI, 0.72-0.80) for the Stockholm3 risk score and 0.60 (95% CI, 0.54-0.65) for the PSA test, both only for men undergoing systematic biopsy. The positive predictive values (PPVs) for detecting clinically significant prostate cancer with a PSA level of 3 ng/mL or higher was 17.6% (106 of 603 patients), 21.3% (145 of 680 patients) with a Stockholm3 risk score of 0.11 or higher, and 28.8% (119 of 413 patients) with a Stockholm3 risk score of 0.15.

## Discussion

In this large, randomized clinical trial, we compared detection of clinically significant prostate cancer using biomarker vs an MRI-enhanced strategy. Specifically, we compared the Stockholm3 risk score plus systematic biopsies against the combination of PSA testing, MRI, and systematic and targeted biopsies. We found no significant difference in the detection of clinically significant prostate cancer between these methods. However, the biomarker-based approach required more biopsies and found more clinically insignificant cancers than the MRI-enhanced strategy. While the Stockholm3 risk score with systematic biopsy strategy can be a suitable alternative in areas with limited MRI access, it may lead to increased numbers of biopsies and detection of less aggressive cancers.

This trial found that both the biomarker-based strategy and the MRI-enhanced approach had similar detection rates for clinically significant prostate cancer. This held true even when considering an alternative definition of clinically significant cancer (a Gleason score ≥4 + 3), with no noteworthy differences in detection between the 2 approaches. When limiting the analysis to systematic biopsies in the MRI-enhanced group, the detection of clinically significant cancer, using either definition, remained similar between the groups. Adherence to the trial protocol varied, with fewer men in the biomarker-based group undergoing biopsy compared with the MRI-enhanced group, possibly due to visible lesions on imaging. However, regardless of the analytic method used, including model-based imputation, the detection of clinically significant cancer aligned with the intention-to-treat analysis.

Prior large trials, including earlier STHLM3-MRI trials, have demonstrated the effectiveness of MRI-targeted biopsy in improving the detection of significant prostate cancer while reducing unnecessary biopsies and overdiagnosis.^2,4,21^ Similarly, the Stockholm3 test has been shown to increase the detection of clinically significant cancer, reduce overdiagnosis, and minimize the number of MRI examinations and biopsies performed.^16,19,22^ However, to our knowledge, this is the first direct comparison between a novel blood-based biomarker and an MRI-enhanced strategy for prostate cancer screening, and our results indicate comparable detection of clinically significant cancer. In prostate cancer screening, there is a need for balance between identifying significant cancer and avoiding harm from unnecessary biopsy procedures and overdiagnosis. While active surveillance has reduced overtreatment, a cancer diagnosis and its surveillance can still cause undue worry and potential harm. In this trial, use of the Stockholm3 test and systematic biopsies instead of an MRI-enhanced strategy would lead to 191 more biopsy procedures and 65 more indolent cancers diagnosed per 10 000 men. The misclassification rate of Gleason scores between the initial biopsy and the final pathology reports was similar between the 2 approaches. While not statistically significant, the biomarker-based approach showed more upgrading and less downgrading of tumors. Since all analyzed biomarkers and their cutoffs displayed similar rates of downgrading and upgrading, this is likely a function of only performing systematic biopsies without MRI.^4^ Downgrading could imply unnecessary treatment, while upgrading might indicate missed cancers.

Prostate biopsies involve a risk of infection. Among men who underwent biopsy, the risk of postbiopsy hospitalization was 3.1% in the biomarker-based group and 3.8% in the MRI-enhanced group. Nevertheless, when comparing postbiopsy hospitalizations for all men between the groups, no significant difference was observed, possibly due to the similar mean number of biopsy cores.

Both MRI-enhanced and biomarker-based strategies have limitations. Diagnostic methods based on MRI findings suffer from low PPV,^11^ high interobserver variability,^10^ and limited access due to cost or a shortage of expertise.^23^ On the other hand, assessment of advanced biomarkers may require expensive laboratory equipment and validation across diverse populations. Site-specific variations in PPV for MRI-targeted biopsies have been reported, with PPV ranging from 19% to 68% for a PI-RADS score of 3 or higher and 26% to 73% for a PI-RADS score of 4 or higher.^11^ In a retrospective single-center study involving multiparametric MRI followed by radical prostatectomy, 16% of clinically significant lesions were missed by MRI readers.^9^ While MRI helps avoid unnecessary biopsies, MRI-targeted biopsies may contribute to overdiagnosis since targeting a suspicious lesion could lead to an upward shift in Gleason score compared with systematic biopsies, and the lesions may not be oncologically equivalent.^12,13,24^ Moreover, certain areas and health care systems may lack the capacity to implement the diagnostic chain required for MRI-based screening. Notably, the cost of MRI of the prostate varies by setting. A US-based study analyzing 552 facilities performing MRI of the prostate found a 26-fold difference in median cost between the least and most expensive facilities.^25^ Patients may also need to travel long distances for an MRI, whereas a blood sample can be sent to a central location for analysis. The implementation of a diagnostic chain, including the Stockholm3 test, is already established in Stavanger, Norway, with 97% of family medicine clinics using it and sending samples to neighboring Sweden for analysis.^26^ While other blood- and urine-based biomarkers also offer higher sensitivity and specificity than PSA for detecting clinically significant prostate cancer, none has been compared with image-based screening. Furthermore, the Stockholm3 test has undergone independent validation, and results from validation in a multiethnic US cohort^27^ and a Central European cohort^28^ show results similar to those in the Scandinavian cohorts. Since the Stockholm3 test is 6 to 7 times more expensive than a PSA test, using PSA as the initial screening criterion is likely necessary to control costs.^29^ Although this study used a 1.5-ng/mL PSA cutoff to initiate further analysis to determine the Stockholm3 risk score, higher cutoffs may optimize cost-effectiveness. A recent analysis suggested that combining the Stockholm3 test with MRI at a PSA threshold of 2 ng/mL or higher was more cost-effective than MRI with a PSA threshold of 3 ng/mL or higher in Sweden.^29^

### Strengths and Limitations

The STHLM3-MRI trial’s primary strengths are its randomized design, large size, and population-based screening setting. Clinical pathways are well controlled to ensure quality MRI and pathological assessments. Nevertheless, limitations include potentially limited generalizability since the study was conducted in Stockholm, Sweden, with centralized review of radiologic and pathological assessments in addition to a homogenous population. The persistence of differences between trial groups in subsequent screening rounds is still being determined. Additional limitations include incomplete data from radical prostatectomies and unknown disease status in men who did not undergo biopsy. Optimal PSA cutoff values for triggering a Stockholm3 test and guiding biopsy decisions are undetermined. Finally, we focused on detecting clinically significant prostate cancer, and long-term prostate cancer mortality implications remain uncertain.

## Conclusions

The findings of this large, randomized clinical trial indicate that a biomarker-based risk prediction approach combining the Stockholm3 risk score with systematic biopsies is comparable with an MRI-enhanced strategy involving PSA levels and both systematic and targeted biopsies for detecting clinically significant prostate cancers as part of a screening program. Nevertheless, the biomarker-based approach comes at the expense of more biopsy procedures and increased detection of less aggressive cancers. In regions where the infrastructure for MRI-based screening is lacking, the Stockholm3 test could aid in selecting men for systematic prostate biopsy.

## Notes

Supplement 1.
Trial Protocol

Supplement 2. **eTable 1.** Relative Proportions of Men with MRI Scans, Biopsies, and Prostate Cancers According to Screening Strategies (Ignoring Biopsy Outcomes for Men in the MRI-Enhanced Group With Negative MRI Results who Underwent Biopsy due to a Stockholm3 Risk Score of 0.25 or Higher) **eTable 2.** Relative Proportion of Men with MRI Scans, Biopsies, and Prostate Cancers According to Screening Strategies (Only Including Men Who Underwent Systematic Biopsies in the MRI-Enhanced Group) **eTable 3.** Gleason Score at Biopsy vs at Prostatectomy in Biomarker-Based Group and MRI-Enhanced Group **eTable 4.** Gleason Score at Systematic Biopsy vs Combined Systematic and Targeted Biopsy for Men in MRI-Enhanced Group who Underwent Both Types of Biopsies **eFigure.** Gleason Score on Systematic Biopsy vs Combined Systematic and Targeted Biopsy for Men in MRI-Enhanced Group who Underwent Both Types of Biopsies

Supplement 3.
Data Sharing Statement

## References

[1] Schröder FH, Hugosson J, Roobol MJ, ; ERSPC Investigators. Screening and prostate cancer mortality: results of the European Randomised Study of Screening for Prostate Cancer (ERSPC) at 13 years of follow-up. Lancet. 2014;384(9959):2027-2035. doi:10.1016/S0140-6736(14)60525-0 25108889 PMC4427906

[2] Kasivisvanathan V, Rannikko AS, Borghi M, ; PRECISION Study Group Collaborators. MRI-targeted or standard biopsy for prostate-cancer diagnosis. N Engl J Med. 2018;378(19):1767-1777. doi:10.1056/NEJMoa1801993 29552975 PMC9084630

[3] van der Leest M, Cornel E, Israël B, . Head-to-head comparison of transrectal ultrasound–guided prostate biopsy versus multiparametric prostate resonance imaging with subsequent magnetic resonance–guided biopsy in biopsy-naïve men with elevated prostate–specific antigen: a large prospective multicenter clinical study. Eur Urol. 2019;75(4):570-578. doi:10.1016/j.eururo.2018.11.023 30477981

[4] Ahdoot M, Wilbur AR, Reese SE, . MRI-targeted, systematic, and combined biopsy for prostate cancer diagnosis. N Engl J Med. 2020;382(10):917-928. doi:10.1056/NEJMoa1910038 32130814 PMC7323919

[5] Eklund M, Jäderling F, Discacciati A, ; STHLM3 Consortium. MRI-targeted or standard biopsy in prostate cancer screening. N Engl J Med. 2021;385(10):908-920. doi:10.1056/NEJMoa2100852 34237810

[6] Mottet N, Cornford P, van den Bergh RCN, ; European Association of Urology; European Association of Nuclear Medicine; European Society for Radiotherapy and Oncology; European Society of Urogenital Radiology; International Society of Urological Pathology; International Society of Geriatric Oncology. Guidelines on prostate cancer. European Association of Urology. Accessed September 25, 2023. https://uroweb.org/guidelines/prostate-cancer/chapter/diagnostic-evaluation

[7] Moses KA, Sprenkle PC, Bahler C, . NCCN Guidelines insights: prostate cancer early detection, version 1.2023. J Natl Compr Canc Netw. 2023;21(3):236-246. doi:10.6004/jnccn.2023.0014 36898362

[8] Wei JT, Barocas D, Carlsson S, . Early detection of prostate cancer: AUA/SUO guideline part II: considerations for a prostate biopsy. J Urol. 2023;210(1):54-63. doi:10.1097/JU.0000000000003492 37096575 PMC11321723

[9] Borofsky S, George AK, Gaur S, . What are we missing? false-negative cancers at multiparametric MR imaging of the prostate. Radiology. 2018;286(1):186-195. doi:10.1148/radiol.2017152877 29053402 PMC5749595

[10] Rosenkrantz AB, Ginocchio LA, Cornfeld D, . Interobserver reproducibility of the PI-RADS version 2 lexicon: a multicenter study of six experienced prostate radiologists. Radiology. 2016;280(3):793-804. doi:10.1148/radiol.2016152542 27035179 PMC5006735

[11] Westphalen AC, McCulloch CE, Anaokar JM, . Variability of the positive predictive value of PI-RADS for prostate MRI across 26 centers: experience of the Society of Abdominal Radiology Prostate Cancer Disease-focused Panel. Radiology. 2020;296(1):76-84. doi:10.1148/radiol.2020190646 32315265 PMC7373346

[12] Vickers A, Carlsson SV, Cooperberg M. Routine use of magnetic resonance imaging for early detection of prostate cancer is not justified by the clinical trial evidence. Eur Urol. 2020;78(3):304-306. doi:10.1016/j.eururo.2020.04.016 32389443 PMC8327360

[13] Vickers AJ. Effects of magnetic resonance imaging targeting on overdiagnosis and overtreatment of prostate cancer. Eur Urol. 2021;80(5):567-572. doi:10.1016/j.eururo.2021.06.026 34294510 PMC8530856

[14] Loeb S, Shin SS, Broyles DL, . Prostate Health Index improves multivariable risk prediction of aggressive prostate cancer. BJU Int. 2017;120(1):61-68. doi:10.1111/bju.13676 27743489 PMC5392379

[15] Bryant RJ, Sjoberg DD, Vickers AJ, . Predicting high-grade cancer at ten-core prostate biopsy using four kallikrein markers measured in blood in the ProtecT study. J Natl Cancer Inst. 2015;107(7):djv095. doi:10.1093/jnci/djv095 25863334 PMC4554254

[16] Grönberg H, Adolfsson J, Aly M, . Prostate cancer screening in men aged 50-69 years (STHLM3): a prospective population-based diagnostic study. Lancet Oncol. 2015;16(16):1667-1676. doi:10.1016/S1470-2045(15)00361-7 26563502

[17] McKiernan J, Donovan MJ, O’Neill V, . A novel urine exosome gene expression assay to predict high-grade prostate cancer at initial biopsy. JAMA Oncol. 2016;2(7):882-889. doi:10.1001/jamaoncol.2016.0097 27032035

[18] Van Neste L, Hendriks RJ, Dijkstra S, . Detection of high-grade prostate cancer using a urinary molecular biomarker-based risk score. Eur Urol. 2016;70(5):740-748. doi:10.1016/j.eururo.2016.04.012 27108162

[19] Nordström T, Discacciati A, Bergman M, ; STHLM3 Study Group. Prostate cancer screening using a combination of risk-prediction, MRI, and targeted prostate biopsies (STHLM3-MRI): a prospective, population-based, randomised, open-label, non-inferiority trial. Lancet Oncol. 2021;22(9):1240-1249. doi:10.1016/S1470-2045(21)00348-X 34391509

[20] Nordström T, Jäderling F, Carlsson S, Aly M, Grönberg H, Eklund M. Does a novel diagnostic pathway including blood-based risk prediction and MRI-targeted biopsies outperform prostate cancer screening using prostate-specific antigen and systematic prostate biopsies? protocol of the randomised study STHLM3MRI. BMJ Open. 2019;9(6):e027816. doi:10.1136/bmjopen-2018-027816 31201191 PMC6576112

[21] Rouvière O, Puech P, Renard-Penna R, ; MRI-FIRST Investigators. Use of prostate systematic and targeted biopsy on the basis of multiparametric MRI in biopsy-naive patients (MRI-FIRST): a prospective, multicentre, paired diagnostic study. Lancet Oncol. 2019;20(1):100-109. doi:10.1016/S1470-2045(18)30569-2 30470502

[22] Ström P, Nordström T, Aly M, Egevad L, Grönberg H, Eklund M. The Stockholm-3 model for prostate cancer detection: algorithm update, biomarker contribution, and reflex test potential. Eur Urol. 2018;74(2):204-210. doi:10.1016/j.eururo.2017.12.028 29331214

[23] Leake JL, Hardman R, Ojili V, . Prostate MRI: access to and current practice of prostate MRI in the United States. J Am Coll Radiol. 2014;11(2):156-160. doi:10.1016/j.jacr.2013.05.006 24389134 PMC4169888

[24] Shoag JE, Cai PY, Gross MD, . Impact of prebiopsy magnetic resonance imaging on biopsy and radical prostatectomy grade concordance. Cancer. 2020;126(13):2986-2990. doi:10.1002/cncr.32821 32320063

[25] Brant A, Wu X, Prunty M, . Variability of prostate MRI charges among U.S. hospital-based facilities. AJR Am J Roentgenol. 2023;220(3):441-442. doi:10.2214/AJR.22.28152 36069483

[26] Viste E, Vinje CA, Lid TG, . Effects of replacing PSA with Stockholm3 for diagnosis of clinically significant prostate cancer in a healthcare system—the Stavanger experience. Scand J Prim Health Care. 2020;38(3):315-322. doi:10.1080/02813432.2020.1802139 32772613 PMC7470071

[27] Vigneswaran HT, Eklund M, Discacciati A, ; SEPTA STHLM3 Study Group. Stockholm3 validation in a multi-ethnic cohort for prostate cancer (SEPTA) detection: a multicentered, prospective trial. J Clin Oncol. 2024;42(4)(suppl):262. doi:10.1200/JCO.2024.42.4_suppl.262 39038251

[28] Elyan A, Saba K, Sigle A, . Prospective multicenter validation of the Stockholm3 test in a central European cohort. Eur Urol Focus. Published online October 7, 2023. doi:10.1016/j.euf.2023.09.016 37813730

[29] Hao S, Heintz E, Östensson E, . Cost-effectiveness of the Stockholm3 test and magnetic resonance imaging in prostate cancer screening: a microsimulation study. Eur Urol. 2022;82(1):12-19. doi:10.1016/j.eururo.2021.12.021 35094896

