# Supplementary material for: Biomarker vs MRI-Enhanced Strategies for Prostate Cancer Screening: The STHLM3-MRI Randomized Clinical Trial
Source: JAMA Netw Open. 2024 Apr 22;7(4):e247131. doi: 10.1001/jamanetworkopen.2024.7131 (PMC11036143; doi:10.1001/jamanetworkopen.2024.7131)
Supplement: Supplement 1. — Trial Protocol [file jamanetwopen-e247131-s001.pdf]

A multicenter randomized diagnostic study assessing whether a diagnostic pathway using the Stockholm3 test and MRI followed by targeted biopsies outperforms PSA and systematic biopsies for men invited to prostate cancer testing.

Acronym: STHLM3 MRI Phase 2

SPIRIT 2013-compliant

STUDY PROTOCOL

V 2.3 2020-01-15

1.1. Table of contents

1.1. Table of contents 2
2. Trial identifier 4
3. Study organization 4
3.1. Principal investigator 4
3.2. Trial steering committee 4
3.3. Trial working group 4
4. Trial Sponsor 4
5. Date and version identifier 5
6. Support 5
7. Abstract 7
8. Introduction 7
8.1. Public health significance of prostate cancer 8
8.2. Early detection and treatment of prostate cancer: benefits and harms 8
8.3. Traditional evaluation of men with increased risk of prostate cancer 9
8.4. Multi-parametric Magnetic Resonance Imaging (mpMRI) for detection of prostate cancer 9
8.5. Targeted prostate biopsies guided by fusion technology 10
8.6. Improving the diagnostic pathway for prostate cancer detection 11
9. Objectives 12
9.1. Primary hypothesis 12
9.2. Additional hypotheses 12
10. Primary aim 14
11. Additional aims 14
12. Study design 14
12.1. Design overview 14
13. Methods: Participants, interventions and outcomes 15

13.1. Study setting 15

13.2. Eligibility criteria 15

13.3. Randomization 15

13.4. Interventions 16

13.5. Outcomes 19

13.6. Follow-up 20

13.7. Serious adverse events 20

13.8. Participant timeline 20

13.9. Sample size 21

13.10. Recruitment and Process Description 21

14. Methods (Data Collection, management, analysis) 24

14.1. Data collection 24

14.2. Data management 24

14.3. Data analysis 24

14.4. Auditing and Monitoring 24

15. Ethics and dissemination 25

15.1. Research ethics approval 25

15.2. Protocol amendments 25

15.3. Consent 25

15.4. Confidentiality 25

15.5. Declarations of interest 25

15.6. Access to data 26

15.7. Dissemination 26

16. Specific considerations 26

Is there a risk of over diagnosis of low risk prostate cancer in men participating in STHLM3-MR? 26

Is there an increased risk of missing aggressive cancers? 26

How do we protect the personal integrity? 26

16.1. Rationale for performing a randomized design in addition to the paired STHLM3-MR and STHLM3 studies 27

17. Authors contribution 27

18. References 27

19. Protocol Addendum STHLM3MR Main Study 31

2. Trial identifier

ClinicalTrials.gov Identifier: NCT03377881

3. Study organization

The co-investigators comprise the principal investigator, the trial steering committee and the trial working group. Decisions on planning or execution of the study, including closure of the study, is to be made by the Principal Investigator alone. The trial steering committee and the trial working group advices the principal investigator.

3.1. Principal investigator

Tobias Nordström, MD PhD

3.2. Trial steering committee

Professor Henrik Grönberg, Martin Eklund, MD PhD

3.3. Trial working group

Markus Aly, MD PhD; Stefan Carlsson, MD PhD; Fredrik Jäderling, MD PhD; Andrea Discaciatti, PhD; Axel Glaessgen, MD PhD

4. Trial Sponsor

Karolinska Institutet, Nobels v12, 17717 Stockholm, Sweden

5. Date and version identifier

Revision<br>version, date, author	Reason	Main Change
V2.1 2018-05-14; TN	After DSMB initial meet	Clarifications regarding working group, and hypotheses
V2.3 2020-01-15	Pre-analysis clarificartions	See separate document

6. Support

This study is supported by grants as outlined below. The study sponsor or funders had no authority over study design, collection, management, analysis or interpretation of data, manuscript drafting, or decision to submit report for publication.

**Grant**	**PI**
Stockholm County Council	Tobias Nordström
Swedish Research Council	Henrik Grönberg
Stockholm City Council	Tobias Nordström
FORTE	Martin Eklund
Erling Persson Foundation	Henrik Grönberg
Swedish Cancer Society	Martin Eklund
Swedish Research Council	Martin Eklund
Karolinska Institutet	Martin Eklund
Prostatacancerförbundet	Martin Eklund

Åke Wiberg Foundation	Martin Eklund

7. Abstract

Prostate cancer is a leading cause of cancer death among men in the Western world. Early detection of prostate cancer has been shown to decrease mortality, but has limitations with low specificity leading to unnecessary biopsies and over-diagnosis of low-risk cancers. The STHLM3 trial has paved the way for improved specificity in early detection of prostate cancer using the blood-based STHLM3 test for identifying men at increased risk of harbouring significant prostate cancer [1].

Targeted prostate biopsies based on MRI images have been shown non-inferior sensitivity to detect significant prostate cancer and decrease the number of biopsies and non-significant cancers among men referred for prostate biopsy in clinical practice [2]. Evidence is limited to study populations including men in current clinical practice.

The overarching strategy of the STHLM3-MR/Fusion projects is to study an improved diagnostic pathway including an improved blood-based test for identification of men with increased risk of prostate cancer and use of MRI to select men for diagnostic workup with targeted prostate biopsies. The aim is to increase the specificity in early detection of prostate cancer without decreasing the sensitivity of aggressive prostate cancers.

Endpoints include the number of detected prostate cancers, number of performed biopsy procedures and number of performed MRIs. Additional aims include to assess the health economic consequences and development of automated image-analysis.

The STHLM3-MR project is performed in two separate phases, analyzed separately. STHLM3-MR Phase 1 is a paired design study which closed inclusion 2017-06-01 and includes 533 men planned for prostate biopsies. All participants underwent target and systematic biopsies together with STHLM3 test analysis. The study constitutes a current practice cohort and levels of the STHLM3 test were not used for selecting participants.

STHLM3-MR Phase 2 is a study comparing traditional prostate cancer detection using PSA and systematic biopsies with the improved pathway for prostate cancer detection using the STHLM3 test and targeted biopsies in a screening context. The study will recruit 10,000 participants during September 2017-April 2018 combining a paired and randomized design. The STHLM3 MRI studies were described previously {NordstromSTHLM3MR} and this protocol follows SPIRIT guidelines [3].

8. Introduction

8.1. *Public health significance of prostate cancer*

Prostate cancer is the most common cancer and the leading cause of cancer death among men in Sweden. In year 2011 over 10,000 men were diagnosed with prostate cancer and more than 2,500 died due to the disease, approximately 20% of these in the Stockholm region. Prostate cancer incidence rates in Sweden are now comparable to rates in countries that had an early introduction of PSA testing, while prostate cancer mortality rates in Sweden are higher than in most other countries[4]. With over 90,000 prevalent cases, the health burden and the costs on the health care system are substantial. While a number of risk factors have been proposed for prevention of prostate cancer, including diet and occupational exposures, the only factors conclusively shown to increase risk of the disease are age, ethnicity and family history. Given the high prevalence of the cancer and limited opportunities for primary prevention, improved detection would reduce both procedure-related harm to men and economical cost in the healthcare system.

8.2. *Early detection and treatment of prostate cancer: benefits and harms*

The PSA test was first used to monitor disease progression in prostate cancer patients. The PSA test was taken up as a *de facto* screening test for prostate cancer in many countries, leading to rapid rises in prostate cancer incidence. The test characteristics for the PSA test in detecting prostate cancer are comparable to those for mammography for breast cancer screening, with a sensitivity of 72% and a specificity of 30-35% at a test threshold of 4 ng/ml[5]. However, a lower threshold of 3 ng/ml adopted in Sweden recently has led to increased sensitivity at the expense of reduced specificity. Recent analyses of PSA testing in the Stockholm area confirms these results showing that 46%, 68% and 77% of men 50-59, 60-69 and 70-79 years respectively have had at least one PSA test during a 9 years period[6].

Recent results from the large European Randomized Study of Screening for Prostate Cancer (ERSPC) including over 180,000 men provide increasing evidence that PSA screening has led to reduced mortality[7]. This report showed that PSA screening without digital rectal examination was associated with a 21% relative reduction in the death rate from prostate cancer at a median follow-up of 11 years, with an absolute reduction of about 7 prostate cancer deaths per 10,000 men screened. Estimations from the ERSPC trial (men aged 55-69) show that 1,048 men would need to be offered screening and an additional 37 would need to be managed to prevent one prostate-cancer death during a 10-year period, leading to a significant overtreatment of indolent disease. The effectiveness of PSA testing was more

marked at the Göteborg site of the ERSPC trial, with a risk reduction of 44% over 14 years in men aged 50-64[8]. This effect size is larger than that observed for mammographic screening for breast cancer and fecal occult blood testing for colorectal cancer.

However, using traditional systematic biopsies for diagnosis, approximately half of diagnosed cancers are low-risk tumors using the same main cutoff for biopsy as the ERSPC trial (PSA=3ng/ml) [9,10]. It has been shown that men with low-risk tumors treated without curative intent have the same survival as men in the background population[11], illustrating the large proportion of over-diagnosed cancers[12].

The STHLM3 study has shown a way to improve identification of men at increased risk of significant prostate cancer. Using the STHLM3 test, 32% of the prostate biopsies may be saved while not decreasing the sensitivity to high-grade disease (defined as Gleason Score ≥7) and simultaneously decreasing the number of low-grade tumors (Gleason Score ≤6) by 17%, thus decreasing overdiagnosis[10].

8.3. Traditional evaluation of men with increased risk of prostate cancer

Men at increased risk of prostate cancer - commonly estimated using PSA and palpatory findings - are traditionally assessed using systematic prostate biopsies. The procedure is performed under local anesthesia using antibiotic prophylaxis and includes 10-12 cores taken from predefined areas of the peripheral zone of the gland as visualized by endorectal ultrasound. While the biopsies systematically covers the prostatic gland rather than targeting a lesion, and non-lethal tumors are common, the risk of over-diagnosis (i.e. detection of non-significant tumors) is high [12]. The risk of non-representative biopsy findings result in underestimation of tumor grade compared with subsequent prostatectomy in up to 40% of men undergoing surgery[13]. The risk of severe post-biopsy infection has increased to 1-2% with increasing frequency of antibiotic resistance, further illustrating the need both to increase precision and decrease the number of performed biopsies[14].

8.4. Multi-parametric Magnetic Resonance Imaging (mpMRI) for detection of prostate cancer

Multi-parametric magnetic resonance imaging (mpMRI) incorporating anatomical and functional imaging has now been validated as a means of detecting and characterizing prostate tumors and can aid in risk stratification and treatment selection. The European Society of Urogenital Radiology (ESUR) in 2012 established the Prostate Imaging Reporting

and Data System (PI-RADS) guidelines aimed at standardizing the acquisition, interpretation and reporting of prostate mpMRI. Consensus on an updated version (PI-RADS v2) have recently been published, outlining aspects of both interpretation and the technical execution[15-17]. Use of the revised PI-RADS provides moderately reproducible MR imaging scores for detection of clinically relevant disease[18]. Using MP-MRI to triage men might allow 27% of patients avoid a primary biopsy and diagnosis fewer clinically insignificant cancers. If subsequent TRUS-biopsies were directed by MP-MRI findings, up to 18% more cases of clinically significant cancer might be detected compared with the standard pathway of TRUS-biopsy for all[19].

In summary, PI-RADS recommends to use 3T or 1.5T machines, including T2- and T1-weighted sequences together with diffusion weighted images (DWI). Currently, the added value of dynamic contrast is not firmly established regarding tumor detection. At this time, there is no consensus among experts concerning the potential benefits of the use of endorectal coils for cancer detection. It has been suggested that the prevalence of suspicious lesions on MRI in men with clinical suspicion of prostate cancer is approximately 60% [20].

8.5. Targeted prostate biopsies guided by fusion technology

Targeted biopsies of the prostate consist of imaging (MRI) detecting significant tumors and a biopsy procedure where biopsies are targeted to the tumor using various devices for guidance [21]. While traditional endorectal ultrasound poorly identifies tumors, direction of biopsy needles can be performed in various ways. Cognitive or soft fusion is based on skilled urologists/radiologists interpreting the MRI images and directing needles solely based on the ultrasound images. The disadvantages of cognitive fusion lie in the potential for human error when attempting to mentally fuse the MRI with TRUS while aiming for cancers that are often <1 cm in diameter and the inability to track the location of each biopsy site. Hard fusion enables proper fusion of MRI information on the ultrasound image, possibly increasing precision.

Despite methodological flaws, a number of studies have investigated the value of fusion biopsies, primarily using non-randomized designs and non-screening populations (see **Error! Reference source not found.**) [22]. In 2018, Kasivisvanathan et al provided high quality evidence for men referred for prostate biopsy and showed that MRI/target biopsies are non-

inferior for detection of significant cancer and decreases the number of in-significant cancers and number of biopsies as compared with systematic biopsies [2].

The proportion of men upgraded when comparing specimen from targeted biopsies and subsequent prostatectomy have been shown to be very low (<5%) when using targeted biopsies[23], increasing the proportion of men where treatment decisions are based on valid risk estimations.

8.6. Improving the diagnostic pathway for prostate cancer detection

The current diagnostic pathway for prostate cancer detection is characterized by several challenging hallmarks. First, testing with PSA is frequent also in men not benefitting from testing due to low PSA levels or high age[6]. Second, the currently used test for detection (PSA) lacks in specificity, resulting in frequent over-diagnosis[24,25]. Third, systematic biopsies shows high frequencies of benign tests, over-diagnosis, up-grading at prostatectomy, and risk of infectious complications[10,26]. Further, PSA testing increases with educational length and men with long education are more likely to have a prostate biopsy after an increased PSA value. These differences may contribute to the worse prostate cancer outcomes observed among men with lower socioeconomic status[27].

The STHLM3 test offers improved disease detection[10]. To further decrease over-detection, improve disease classification and spare men of test-related harm, prostate biopsy practice need to be improved. We hypothesize that an improved pathway for prostate cancer detection including a better blood-based screening test, improved selection to biopsy based on MRI findings and targeted biopsies guided by MRI/ultrasound fusion would dramatically decrease the number of biopsy procedures, overdiagnosis and improve treatment decisions (see Figure 1).

Figure 1: Improved pathway for prostate cancer detection.

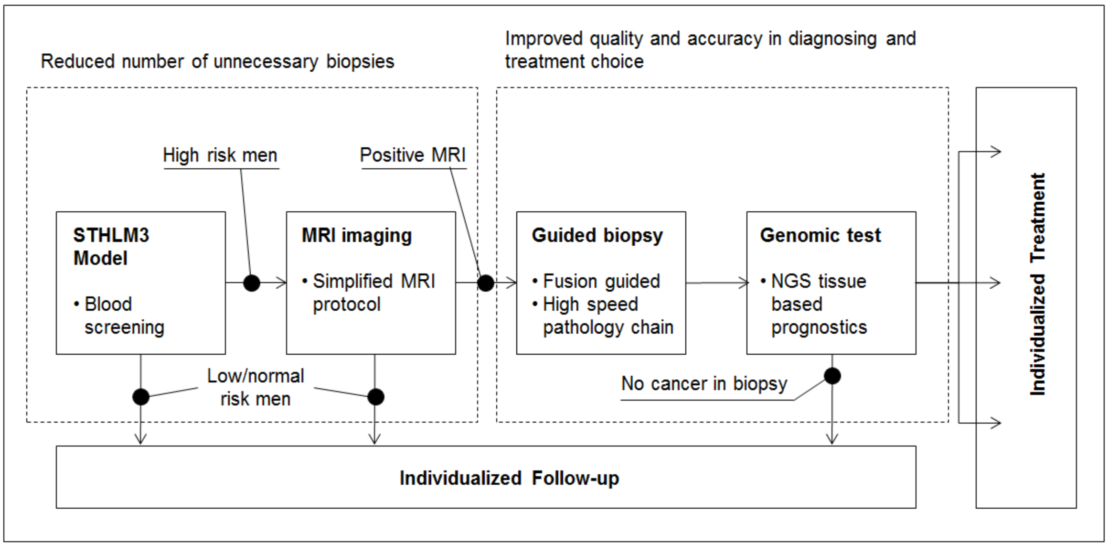

9. Objectives

9.1. Primary hypothesis

The overarching primary hypothesis of the STHLM3MRI trial is that a diagnostic pathway using the Stockholm3 test to select men for further workup using MRI followed by targeted biopsies and systematic biopsies (S3M+MRI+TBx+SBx) has non-inferior sensitivity for detecting clinically significant cancer (ISUP grade ≥2) and shows superior specificity (reduction in number of performed biopsy procedures and detected ISUP 1 tumours) compared with the diagnostic pathway using systematic biopsies in men with PSA ≥ 3 ng/mL (PSA+SBx).

9.2. Additional hypotheses

1. When compared with performing systematic biopsies for men with elevated risk of prostate cancer in prostate cancer screening, targeted and systematic prostate biopsies performed on MRI positive men has non-inferior sensitivity for detecting clinically significant cancer (ISUP grade group ≥2) and reduces the number of performed biopsy procedures, which also translates to lower proportion of men with elevated risk who experience severe post-biopsy infections. Elevated risk can here be defined using PSA or S3M – we will clarify the exact contrasts for testing this hypothesis below.
2. When compared with performing systematic biopsies for men with elevated risk of prostate cancer in prostate cancer screening, targeted biopsies only

performed on MRI positive men has non-inferior sensitivity for detecting clinically significant cancer (ISUP grade group ≥2) and reduces the number of performed biopsy procedures, which also translates to lower proportion of men with elevated risk who experience severe post-biopsy infections. Elevated risk can here be defined using PSA or S3M – we will clarify the exact contrasts for testing this hypothesis below.

3. A diagnostic chain consisting of Stockholm3 followed by MRI and targeted+systematic biopsies (S3M+MRI+TBx+SBx) versus a diagnostic chain based on PSA ≥3 ng/ml followed by MRI and targeted+systematic biopsies (PSA+MRI+TBx+SBx) will lead to: non-inferior sensitivity for detecting clinically significant cancer (ISUP grade group ≥ 2); an inferior sensitivity for ISUP1 cancers (i.e. reduced overdiagnosis); and a reduction in the number of MRI examinations and performed biopsies.
4. A diagnostic pathway using the Stockholm3 test to select men for further workup using MRI followed by ONLY targeted biopsies (S3M+MRI+TBx) has non-inferior sensitivity for detecting clinically significant cancer (ISUP grade group ≥2) and reduces the number of performed biopsy procedures compared with a diagnostic pathway using systematic biopsies in men with PSA ≥3 ng/mL (PSA+SBx).
5. Biopsy compliance is higher after biopsy is recommended based on MRI compared to recommended without MRI.
6. SBx in the MRI arm has superior sensitivity than SBx in the non-MRI arm (due to cognitive fusion).
7. A diagnostic chain consisting of Stockholm3 followed by MRI and targeted+systematic biopsies (S3M+MRI+TBx+SBx) is cost-effective (ICER < 750 000 SEK per QALY gained) compared to a diagnostic chain based on PSA ≥3 ng/ml followed by MRI and targeted+systematic biopsies (PSA+MRI+TBx+SBx).
8. A diagnostic chain using the Stockholm3 test to select men for further workup using MRI and targeted+systematic biopsies (S3M+MRI+TBx+SBx) is cost-effective compared to a diagnostic chain using systematic biopsies in men with PSA ≥3 ng/ml (PSA+SBx).
9. Adding prostate volume as a variable in the diagnostic chain with Stockholm3 test (i.e. using the full Stockholm3 model described in Ström et al.[5]) and MRI/Fusion biopsies improves model precision, leading to further improvements

in specificity compared to the use of the Stockholm3 test without the inclusion of prostate volume.

10.Primary aim

To compare a diagnostic pathway using the Stockholm3 test to select men for further workup using MRI (PI-RADS ≥ 3) and targeted biopsies (S3M+TBx) to a diagnostic pathway using systematic biopsies in men with PSA ≥3 ng/ml (PSA+SBx) with respect to number of diagnosed clinically significant cancer (ISUP grade group ≥ 2) and number of performed biopsies.

11.Additional aims

Additional aims corresponding to hypotheses 2-9 above will be assessed.

12. Study design

12.1. Design overview

STHLM3-MR Phase 2 is a study combining a paired and a randomized design (see below). The study will follow the following outline: Participants will be invited by mail. All participants will undergo a blood-test, including PSA and the STHLM3 test. Men with an elevated PSA ≥3 ng/ml *or* PSA ≥1.5ng/ml and S3M>11% will be randomized to either traditional prostate biopsies or MR with targeted biopsies on MR lesions.

Figure 2: Design overview

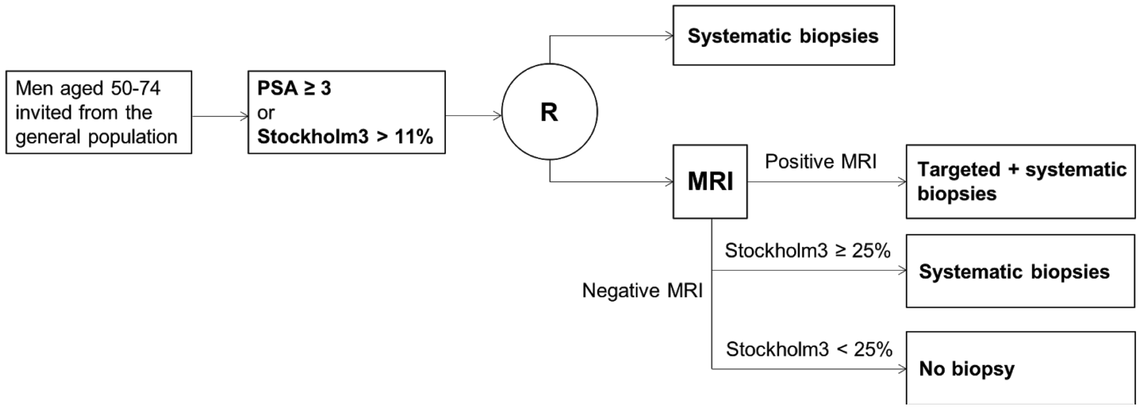

13. Methods: Participants, interventions and outcomes

13.1. Study setting

This is a screening-by-invitation study including one study administrative center, two radiological sites and three urological sites where data will be collected.

Participating urological centras

Department of Urology, Capio St Görans Hospital: dr Henrik Grönberg
Uroclinic, Sophiahemmet, Stockholm; dr Olof Jansson
Odenplans läkarhus; dr Magnus Annerstedt
Urologifocus; dr Gunnar Trygg

13.2. Eligibility criteria

Inclusion criterias

Men age 50-74 years without prior diagnosis of prostate cancer (ICD-9 C61).

Permanent postal address in Stockholm

Not a previous participant in the Stockholm3 study (2012-2014)

Exclusion criterias

Severe illnesses such as metastatic cancers, severe cardio-vascular disease or dementia

Contraindications for magnetic resonance imaging (MRI) eg pacemaker, magnetic cerebral clips, cochlear implants or severe claustrophobia.

Men with a previous prostate biopsy the preceding 60 days before invitation.

13.3. Randomization

Randomization is performed 2:3 between control arm and experimental arm. Randomisation will be performed using stratification on disease risk (three strata). Disease risk is assessed using the Stockholm3 test.

Six allocation lists have been created, specifying the sequence of study arm allocation. Participants are first allocated to corresponding list, and then allocated to study arm

according to the order in which they participate. The allocation sequence is blinded from the study investigators and handled by the study database administrator (SDA, A Björklund).

In order to enhance resource usage, men are allocated to the study sites according to local availability of biopsy procedure slots.

13.4. Interventions

Blood sampling

Participating men undergo blood-sampling with analysis of PSA and the Stockholm3 test at any of Unilabs blood-testing site .

For the main analysis, the Stockholm3 test include clinical data as answered when consenting participation (previous biopsy, age, finasteride medication, relatives with prostate cancer); single nucleotide polymorphisms and measurements of protein levels (MSMB, MIC1, PSA, fPSA, hK2). For secondary analyses, clinical information on DRE and prostate volume is included. The algorithm for calculation of the Stockholm3 test result has been described (Ström et al, European Urology 2018).

Definition of EXPERIMENTAL ARM

Men randomized to the experimental arm undergoes MRI. If suspicious lesions are found, the participant undergoes targeted biopsies using Fusion technology *followed by systematic biopsies.*

Men without lesions are excepted from further intervention and receives notification on recommendation for follow-up. Technology and process are described below.

Men with a Stockholm3 risk ≥25% and no suspicious lesion on MRI will undergo systematic biopsies.

Definition of CONTROL ARM

Men randomized to the control arm undergoes systematic biopsies as defined below.

Technology

Cut-offs for performing the STHLM3 test

The STHLM3 test will be performed for men with a PSA ≥ 1.5 ng/ml

Cut-offs for entering randomization

Participants with PSA ≥ 3.0 ng/ml or STHLM3-test ≥ 11% risk of Gleason Score ≥7 cancer will be randomized and offered to undergo either MR or systematic biopsies (See Process description).

MRI technology

Location and MRI equipment

Capio St Görans Hospital: General Electric 3T

Globen Healthcare: Siemens Magnetom Aera 1.5T

Patient preparations

Refraining from sexual activity with ejaculation 3 days prior to examination

Fasting patient 6 h

Minimal preparation enema prior to examination

Antispasmodic agent (Glucagon) just before the examination

MRI Protocol

A short (16 minutes) MRI protocol developed through STHLM3MR Phase 1 will be used. A detailed description of protocols used below. Briefly, the protocol includes: 3D T2 alt T2 ; Diffusion for ADC B100 , B450, B800, B1500 limited to the prostate location; T1 or FS limited to the prostate location; No endorectal coil will be used.

Image processing the ADC map is fusioned together with the T2 series using Nordic Ice software. The Color scale for ADC maps is the inverted rainbow color scale. Color adjustment is set to min. 50 and max. 220.

Definition of MRI protocol:

MRI Protocol, 1,5 T Siemens Magnetom Aera						
Sequence, plane of acquisition	Pulse sequence	Repetition time/Echo time (ms)	Acquired voxel size (mm)	Field of view (mm)	Slices (n)	Time of acquisition (min:sec)
3 Plane Localizer	FSE	1500/102	1.8x1.8x8	460 x 460		00:20
T2w 2D ax	TSE	3200/134	0.6x0.6x3	200 x 200	30	03:20
T2w 2D, sag	TSE	3630/117	0.6x0.6x4	265 x 215	13	1:40
T2w 2D, cor	TSE	3250/134	0.6x0.6x3	200 x 200	16	01:42
T1w 2D, ax	Vibe	6.86/2.38/4.75	0.6x0.6x3	380 x 297		00:20
DWI (focus), ax, b=0,800	EPI DWI	4140/57	2x2x4	200 x 200	22	03.41

MRI protocol, 3T Signa Architect, GE Healthcare						
Sequence, plane of acquisition	Pulse sequence	Repetition time/Echo time (ms)	Acquired voxel size (mm)	Field of view (mm)	Slices (n)	Time of acquisition (min:sec)
3 Plane Localizer	SE	Minimum /80		420 x 420		00:17
T2w 2D ax	FSE	3000/120	0.6x0.6x3.0	200 X 200	32	04:36
T2w 2D, sag	FSE	2500/120	0.6x0.6x3.0	180 X 180	24	02:05
T2w 2D, cor	FSE	2500/120	0.6x0.7x3.5	200 x 200	24	02:05
T1w 2D, ax	FSE	767/Minimum Full	0.9x1.1x4.0	250 x 250	20	00:35
DWI (focus), ax, b=0,1000*	DWI	4996 / Minimum	1.7x1.7x4.1	200 x 100	17	03:45

MRI Interpretation

MRI interpretation is centralized to Capio St Görans hospital. Assessments are based on "Assessment Without Adequate DCE" from PI-RADS v2 and v2.1.

Dr Fredrik Jäderling is responsible for MRI interpretation. Dr Jäderling or 1-2 other, experienced radiogists at his department performs all MRI interpretations.

Fusion biopsy technology

Brand/models

BK Medical (BK Ultrasound ; www.bkultrasound.com/bk-medical/fusion)

The BK Medical fusion system is the only fusion device compatible with BK Medicals ultrasound devices, used by the urology departments participating in the study. The system represents a second generation ultrasound system with integrated MRI Fusion. MRI data is imported through HIPAA-compliant PACS connection with the local radiology department.

Definition of targeted biopsies

Using MRI data with pre-marked borders of the prostate and tumor, fusion of MRI images and ultrasound images are performed bedside. Using local anesthetic and antibiotic prophylaxis, lesions are according to below. Targeted biopsies are always combined with systematic biopsies.

Biopsy procedure for targeted biopsies

PI-RADS≥3: 3-4 targeted biopsies on marked lesions + systematic biopsies

Large diffuse lesions or poor image quality: Systematic biopsies including lesion

No PI-RADS≥3, diffuse lesions and at least acceptable image quality: No biopsies are performed.

Definition of systematic biopsies

10-12 systematic biopsies are taken from the peripheral zone as previously described in STLHLM3 and the National Guidelines. Extra biopsies are allowed from additional sites visible on ultrasound or according to palpatory findings. In summary, systematic biopsies are performed in the peripheral zone as 4 lateral and para-median biopsies on the left and right side, in the base and mid part of the gland. In the apical third of the gland one lateral left and right biopsy is performed.

Pathology

Pathology is centralized to Unilabs/Capio St Görans hospital. Dr Axel Glaessgen is responsible for the integrity of analyzes of pathological specimen. 2-3 uro-pathologists at dr Glaessgens department assesses all pathological specimen with intermittent cross-validation between them. Pathology preparation and reporting follow ISUP 2014 guidelines.

The pathology preparation is done by Unilabs as part of the normal clinical routine. Biopsy specimens are analyzed according to local practice.

Localisation of biopsies in the prostate are described using Swedish National Guideline nomenclature (A1-4; B1-4; C1-4; anterior/posterior). Gleason Score, mm cancer and % Gleason 4 is reported on each needle specimen.

Pathologist notes results in the usual way in the laboratory system. The result of the pathological analysis is submitted in accordance to existing clinical routines to the referring urologist. A copy of the result is delivered to the study administration.

13.5. Outcomes

Primary otcome:

1. Diagnosed ISUP grade ≥ 2 cancers

Key secondary outcomes:

2. Diagnosed ISUP grade 1 cancers
3. Performed biopsies
4. Performed MRI examinations

See statistical analysis plan (SAP) for details.

Additional endpoints

We are collecting data on a large number of endpoints in the study. See SAP for detailed information regarding the definition of these endpoints.

13.6. Follow-up

All participants were followed a minimum of 200 days after receiving blood test results. Main study outcomes are assessed after prostate biopsy procedures (plus 30 days of follow-up for post-biopsy infections). Additional participant data will be secured in the following circumstances:

No suspicious lesion on MRI:

Men in the experimental arm without suspicious lesions on MRI will be informed and recommended follow-up by the responsible, local urologist. After additional ethical application, the co-investigators might initiate retrospective follow-up of these participants.

Men with diagnosed prostate cancer

Participants with prostate cancer diagnosed on biopsy within the study will be followed up after the biopsy to secure data on the following: Treatment modality (Active Surveillance, Surgery, Radiation); Treatment lead-time and site; Pathological report after surgery (positive margins, T-stage, etc). Data will be assessed through medical records intermittently.

13.7. Serious adverse events

Study nurse will monitor serious adverse events after the prostate biopsy procedures (up to 30 days post biopsy). To ensure this, the study nurse will check medical journals for hospitalization within 1 week after the biopsy procedure in the journal systems Take Care and Cosmic (covering the main part of hospitals in Stockholm region). This will be initiated as individual biopsy results are registered at the study administration. Results will be provided to the Data Safety and Monitoring Board.

13.8. Participant timeline

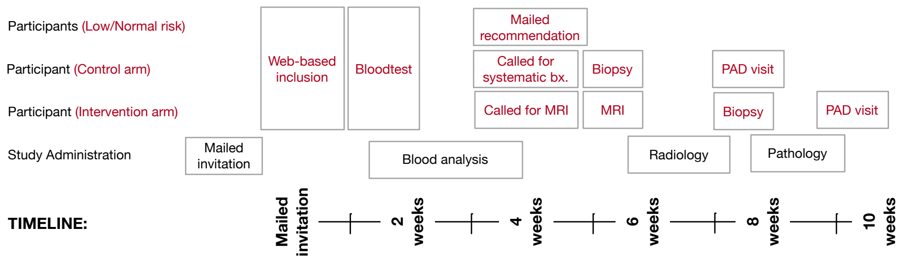

13.9. *Sample size*

Sample size calculations are described in the Statistical Analysis Plan (SAP).

13.10. *Recruitment and Process Description*

The STHLM3-MR Phase 2 will use existing solutions developed and optimized in the previous studies STHLM3 and STHLM3-MR Phase 1 where all major components of the process have been tested.

Figure 2: STHLM3 MR Phase II: Overall design and main process steps. First, participants will follow the *paired design study process* where inclusion, blood-test and delivery of recommendation letter is performed. Men with increased risk of high-grade prostate cancer then enter the *randomized study process*, where extended work-up including biopsies are performed.

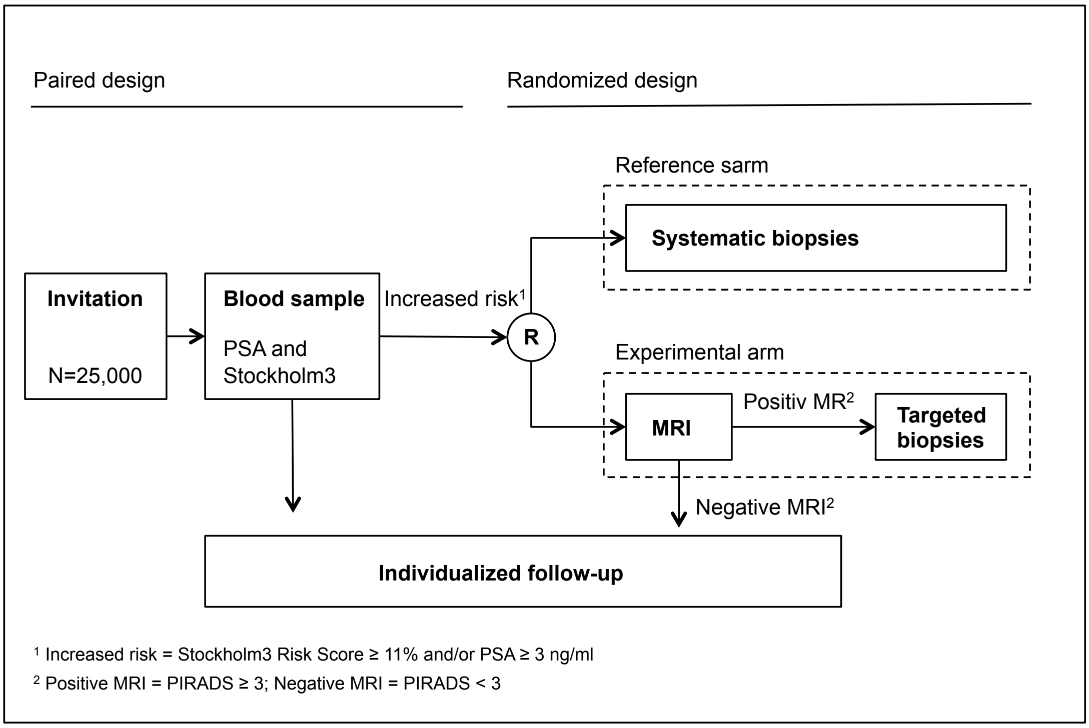

Study part 1 (All participants): Paired study process

Step 1: Selection and Invitation

A potential participant list is created by collecting names and addresses for men between ages 50-74 with a permanent mailing address in Stockholm county. Name and address details of potential participants are bought from an external address source, such as SPAR or InfoTorg.

The address list of potential participants is loaded into STHLM3 Participant Handling Register (Microsoft Dynamics CRM configured for this purpose). For each potential participant a unique study ID is created.

An invitation letter is sent to each potential participant, asking if they are willing to participate in the STHLM3 MR Phase II study. The invitation includes:

1. Invitation letter with brief information about the study, including what the potential participant need to do in order to participate
2. Study information brochure with extensive information on the study
3. List of about 60 laboratories in Stockholm that participate in the study including addresses, opening hours, phone numbers, etc.
4. The proposed participant is directed to the website medicinskastudier.se for inclusion. At this website, a secure login using "Mobilt BankID" is used and the participant is included after answering studyrelated questions (family history, previous prostate biopsy and current use of selected medicine (finasterid, avodart, dutasterid, proscar and testosteron)). Informed consent is acquired and a unique Study ID is created.
5. An electronic referral with a unique referral ID (RemissID) is generated and activated for blood sampling at any of the blood-sampling stations.

Step 2: Blood Sampling

The research participants who choose to join the study visit one of the 60 laboratories that collaborate with STHLM3. For many participants, this means that sampling can be conducted close to home or work.

The lab personnel first check the research participant's identity. This is done in the normal way, i.e. by checking the man's identity card and checking that the photo matches the man. The lab personnel then scan the following information on the combined referral and consent form: RemissID, laboratory analysis code (=code that steers robots and transport routines at the laboratory, barcoded) and Kombika (=code for automatic payment through the health care system, barcoded).

The lab personnel then samples a Stockholm3 test from the participant. This is normal venous blood samples of 12 ml.

The blood tests are then passed through the regular health care logistics to A23 laboratory for analysis. A23 conducts the Stockholm3 analysis and sends the results together with the referral ID (RemissID) to the STHLM3 research group. KUL also sends the combined referral and consent form to the STHLM3 research group.

Step 3: Randomization

Research participants with a total PSA ≥ 3 ng/ml and/or a Stockholm3 risk score ≥ 11% (see above) will be randomized 2:3 to either the reference arm with traditional, systematic biopsies, or to the experimental arm with MRI followed by targeted biopsies.

4: Response letters to the participant

Each participant is placed in a group based on the test results:

Green: PSA < 1.5

Yellow: 1.5 ≤ PSA < 3 and Stockholm3 risk score < 11%

Red: PSA ≥ 3 and/or Stockholm risk score ≥ 11%

Each participant will receive any of four response letters (snail mail) within 5 weeks of sampling. The response letter is sent to the participants' registered permanent home address:

Green: Low risk for prostate cancer, follow-up test within 6-10 years is recommended. For participants > 60 years of age, no more testing is recommended

Yellow: Normal risk for prostate cancer, follow-up within 2-4 years is recommended

Men with increased risk of prostate cancer will enter the randomized part of the study. They receive letters according to study arm:

Red (reference arm): Increased risk of prostate cancer, urology consultation and biopsy is recommended. The biopsies will be performed using traditional technique.

Red (experimental arm): High risk of prostate cancer and extended work-up is recommended. The participant will be referred for MRI examination and subsequent visit to a participating urology office where targeted biopsies will be completed to suspicious lesions. If no lesions are detected, the urologist will recommend structured follow-up.

A list with participants with high risk (Red) are created weekly and delivered from the study administration to the participating urology department, including information on STHLM3 risk (%), PSA, responsible urology department (St Göran , Odenplan, Sophiahemmet) and study arm (experimental, control)

Participants are contacted from each department to book time for MRI (experimental arm) or systematic biopsy).

The participant that choses to continue with urology visit or MRI will become patients in the normal Swedish health care system, i.e. tax paid health care.

Step 5a: Reference arm (Traditional biopsies)

Men randomized to the reference arm will be referred for systematic biopsies at any of the participating urology centers according to Definition of systematic biopsies

After performed biopsy, men are followed in line with clinical practice.

STEP 5b: Experimental arm (MRI and targeted biopsies)

Men randomized to the experimental arm will be referred to MRI at S:t Göran Hospital or at Unilabs Globen. Radiology data is transferred in accordance to clinical practice to Karolinska Hospital or Capio St Görans radiology department and evalutated in accordance to PI-RADS v2 by study radiologists. Suspected lesions are marked and the report is transmitted to S:t Görans Hospital.

Men with PI-RADS≥3 lesions will undergo targeted biopsies followed by systematic biopsies. MRI data is loaded into the local Fusion software by the responsible urologist at the time of the biopsy procedure. All procedures include local anestethics and antibiotic

prophylaxis as recommended by National Guidelines. Separate referrals for systematic and targeted biopsies are used (see appendix).

Men with no visible PI-RADS≥3 lesions will be informed by participating urologist and given instructions for systematic follow-up. This follow-up is recommended to include a renewed MR and a STHLM3 test after 12 months.

14. Methods (Data Collection, management, analysis)

14.1. Data collection

Primary data sources are

i. clinical variables collected from laboratory referral
ii. biopsy referrals and reports
iii. pathology reports
iv. MRI reports
v. blood analysis reports

Collection of i. – iv. is performed by study nurses (C Cavalli-Björkman) on a weekly basis from participating urology sites, participating radiologists. For v., this is digitally transferred from A23 laboratory.

14.2. Data management

Data is collected, entered, coded and stored at Department of Medical Epidemiology and Biostatistics, Karolinska Institutet. Data is entered by Study Nurse using predefined database sheets. This is blinded from study co-investigators and data is stored at the department under supervision by the study database administrator (SDA, Astrid Björklund). Any extraction of study data is performed by the SDA after approval of PI Tobias Nordström.

14.3. Data analysis

Analysis of data is described in the Statistical Analysis Plan (SAP).

14.4. Auditing and Monitoring

A Data Safety and Monitoring Board (DSMB) is assembled and consist of dr Hans Garmo (Statistician), prof Ola Bratt (Urology) and prof Holmberg (Urology/Study Design). The DSMB audits protocol and process descriptions and interim data extraction performed by the study database administrator and the study statistician (AD). The co-investigators are blinded to

the interim data and analysis results. The work of the DSMB is regulated in the DSMB Charter.

15. Ethics and dissemination

15.1. Research ethics approval

The study has approval from the regional ethical board in Stockholm (2017-1280/31).

15.2. Protocol amendments

Minor changes to this protocol made after 2018-04-04 is noted in the protocol. Major changes including changes to eligibility criteria, outcomes, analyses are registered at clinicaltrials.gov and communicated to the DSMB for recommendations on further disseminations.

15.3. Consent

Participant consent is secured when the participant is included to the study at www.kliniskastudier.se. This includes secure identification using Mobilt BankID. Additional approval on use of biological specimen data is collected on the biopsy referral.

15.4. Confidentiality

Study data is collected and stored at Department of Medical Epidemiology and Biostatistics, Karolinska Insitutet using secure Oracle servers. All data extractions are made by database administrator and are anonymized (personal id number is removed) before dissemination to researchers.

15.5. Declarations of interest

Henrik Grönberg has five prostate cancer diagnostic related patents pending, has patent applications licensed to Thermo Fisher Scientific, and might receive royalties from sales related to these patents. Martin Eklund is named on four of these five patent applications. Karolinska Institutet collaborates with Thermo Fisher Scientific in developing the technology for the Stockholm3 test.

15.6. Access to data

Co-investigators will have access to the anynomized, final data-set. Publication of any post-hoc analyses are permitted after communication with Tobias Nordström or by him delegated person. The data-set might be accessible for external validation of trial results on communication with Tobias Nordström.

15.7. Dissemination

Analyses results on the posed aims will be submitted for peer-reviewed publication and submitted for presentation at scientific congress. Communication of the results will be made to patient organisations (Prostatacancerförbundet) and non-scientific channels. No use of professional writers are planned.

The study protocol is made publicly availiable through clinicaltrials.gov.

16. Specific considerations

Is there a risk of over diagnosis of low risk prostate cancer in men participating in STHLM3-MR?

There is a risk of over-diagnosis when performing systematic biopsies of the prostate, which is the current golden standard in clinical practice. While targeted biopsies are performed only towards visible lesions on MRI, and the problem of up-grading is substantially lower in targeted biopsy cohorts, the risk of causing over-diagnosis using targeted biopsies are low.

Is there an increased risk of missing aggressive cancers?

The sensitivity of finding high-grade cancers is very limited in current clinical practice, using PSA with a poorly defined cut-off and non-targeted, systematic biopsies for extended diagnostic work-up. Depending on the cut-off used for recommending biopsy (e.g. STHLM3-test cutoff and MR-cutoff using PIRADS-grade) this sensitivity can be adjusted. Previous studies show that the sensitivity/specificity balance of the STHLM3-test is better than PSA and that this balance is better for targeted biopsies than traditional biopsies. Using these individually superior components, we aim to keep the sensitivity to high-grade cancers stable, while improving specificity significantly. Thus, a similar number of aggressive cancers will be detected, but hopefully to a lower cost in terms of biopsies performed and un-harmful disease detected.

How do we protect the personal integrity?

Information obtained in the study will be collected in one database. The purpose of the database is to collect study data in a proper and safe way for a long time. All information about the participants will be treated with utmost confidentiality and with

strong safeguards to preserve their anonymity. Information that can be used to identify the participant (such as name, address and social security numbers) is always kept separate from other data (such as survey responses and blood tests). All questionnaire data and test results will be treated to prevent unauthorized access to them. The samples will get a unique code so that outsiders cannot identify them. The participants' samples are treated in accordance with the Swedish Biobank Act.

Everyone who works with STHLM3 are under confidentiality agreements. Results from the study are presented only as statistics in which individual answers cannot be traced.

Treatment of the personal data is in accordance with the Swedish Personal Data Act (1998:204). All participants may request in writing to find out what information about them self from which information has been collected and to whom the data has been disclosed. STHLM3's adherence to the Swedish Personal Data Act has been reviewed by the Swedish Data Inspection Board. A preliminary decision has been issues (DNR 1278-2012).

16.1. Rationale for performing a randomized design in addition to the paired STHLM3-MR and STHLM3 studies

	**Strengths**	**Weaknesses**
**Randomized design**<br><br>**(Phase 2)**	• **Study test entire diagnostic pathway prospectively in one study**<br>• Minimizes bias and contamination<br>• Compares fusion bx to "existing gold standard".<br>• Current practice biopsies performed at network urologist<br>• Only 50% of participants undergoes MRI.<br>• Studies screening population in contrast to population coming for biopsy in current clinical practice (selection).	• Increased participant nr.<br>• Increased costs (STHLM3 tests, biopsies)<br>• Screening population, i.e. lower mean PCa risk, smaller mean tumor size

17. Authors contribution

TN was the Principal Investigator. TN, HG, ME, SC and MA designed the study. ME and TN interpreted preliminary data. FJ designed MRI protocols and collected data.

18. References

1. Grönberg H, Adolfsson J, Aly M, et al. Prostate cancer screening in men aged 50-69 years (STHLM3): a prospective population-based diagnostic study. Lancet Oncol 2015;16(16):1667–76.

2. Kasivisvanathan V, Rannikko AS, Borghi M, et al. MRI-Targeted or Standard Biopsy for Prostate-Cancer Diagnosis. N Engl J Med 2018;378(19):1767–77.

3. Chan A-W, Tetzlaff JM, Altman DG, et al. SPIRIT 2013 statement: defining standard protocol items for clinical trials. 2013. p. 200–7.

4. Ferlay J, Soerjomataram I, Dikshit R, et al. Cancer incidence and mortality worldwide: Sources, methods and major patterns in GLOBOCAN 2012. Int J Cancer 2014;:n/a–n/a.

5. Mistry K, Cable G. Meta-analysis of prostate-specific antigen and digital rectal examination as screening tests for prostate carcinoma. J Am Board Fam Pract 2003;16(2):95–101.

6. Nordström T, Aly M, Clements MS, Weibull CE, Adolfsson J, Grönberg H. Prostate-specific antigen (PSA) testing is prevalent and increasing in Stockholm County, Sweden, Despite no recommendations for PSA screening: results from a population-based study, 2003-2011. Eur Urol 2013;63(3):419–25.

7. Schröder FH, Hugosson J, Roobol MJ, et al. Prostate-cancer mortality at 11 years of follow-up. N Engl J Med 2012;366(11):981–90.

8. Hugosson J, Carlsson S, Aus G, et al. Mortality results from the Göteborg randomised population-based prostate-cancer screening trial. Lancet Oncol 2010;11(8):725–32.

9. Nationell kvalitetsrapport för diagnosår 2012 [Internet]. Regionala Cancercentrum i Samverkan; 2013. Available from: http://npcr.se/wp-content/uploads/2013/04/20131121-NPCR-Rapport-2012.pdf

10. Grönberg H, Adolfsson J, Aly M, et al. Prostate cancer screening in men aged 50-69 years (STHLM3): a prospective population-based diagnostic study. Lancet Oncol 2015;16(16):1667–76.

11. Rider JR, Sandin F, Andrén O, Wiklund P, Hugosson J, Stattin P. Long-term outcomes among noncuratively treated men according to prostate cancer risk category in a nationwide, population-based study. Eur Urol [Internet] 2013;63(1):88–96. Available from: http://www.sciencedirect.com.proxy.kib.ki.se/science/article/pii/S0302283812009256/pdfft?md5=babc39ae400d067768cc13a694e3925b&pid=1-s2.0-S0302283812009256-main.pdf

12. Loeb S, Bjurlin MA, Nicholson J, et al. Overdiagnosis and overtreatment of prostate cancer. Eur Urol 2014;65(6):1046–55.

13. Soares R, Di Benedetto A, Dovey Z, Bott S, McGregor RG, Eden CG. Minimum 5-year follow-up of 1138 consecutive laparoscopic radical prostatectomies. BJU Int 2015;115(4):546–53.

14. Aly M, Dyrdak R, Nordström T, et al. Rapid increase in multidrug-resistant enteric bacilli blood stream infection after prostate biopsy-A 10-year population-based cohort study. Prostate 2015;75(9):947–56.

15. Barrett T, Turkbey B, Choyke PL. PI-RADS version 2: what you need to know. Clin Radiol 2015;70(11):1165–76.

16. Barentsz JO, Weinreb JC, Verma S, et al. Synopsis of the PI-RADS v2 Guidelines for Multiparametric Prostate Magnetic Resonance Imaging and Recommendations for Use. Eur Urol 2015;69(1):41–9.

17. Weinreb JC, Barentsz JO, Choyke PL, et al. PI-RADS Prostate Imaging - Reporting and Data System: 2015, Version 2. Eur Urol 2015;69(1):16–40.

18. Muller BG, Shih JH, Sankineni S, et al. Prostate Cancer: Interobserver Agreement and Accuracy with the Revised Prostate Imaging Reporting and Data System at Multiparametric MR Imaging. Radiology 2015;277(3):741–50.

19. Ahmed HU, El-Shater Bosaily A, Brown LC, et al. Diagnostic accuracy of multi-parametric MRI and TRUS biopsy in prostate cancer (PROMIS): a paired validating confirmatory study. Lancet 2017;389(10071):815–22.

20. Moore CM, Robertson NL, Arsanious N, et al. Image-guided prostate biopsy using magnetic resonance imaging-derived targets: a systematic review. Eur Urol 2013;63(1):125–40.

21. Sonn GA, Margolis DJ, Marks LS. Target detection: magnetic resonance imaging-ultrasound fusion-guided prostate biopsy. Urol Oncol 2014;32(6):903–11.

22. Schoots IG, Roobol MJ, Nieboer D, Bangma CH, Steyerberg EW, Hunink MGM. Magnetic Resonance Imaging-targeted Biopsy May Enhance the Diagnostic Accuracy of Significant Prostate Cancer Detection Compared to Standard Transrectal Ultrasound-guided Biopsy: A Systematic Review and Meta-analysis. Eur Urol 2014;68(3):438–50.

23. Baco E, Ukimura O, Rud E, et al. Magnetic resonance imaging-transectal ultrasound image-fusion biopsies accurately characterize the index tumor: correlation with step-sectioned radical prostatectomy specimens in 135 patients. Eur Urol 2015;67(4):787–94.

24. Arnsrud Godtman R, Holmberg E, Lilja H, Stranne J, Hugosson J. Opportunistic Testing Versus Organized Prostate-specific Antigen Screening: Outcome After 18 Years in the Göteborg Randomized

Population-based Prostate Cancer Screening Trial. Eur Urol 2014;68(3):354–60.

25. Thompson IM, Pauler DK, Goodman PJ, et al. Prevalence of prostate cancer among men with a prostate-specific antigen level. N Engl J Med 2004;350(22):2239–46.

26. Loeb S, Vellekoop A, Ahmed HU, et al. Systematic review of complications of prostate biopsy. Eur Urol 2013;64(6):876–92.

27. Nordström T, Bratt O, Örtegren J, Aly M, Adolfsson J, Grönberg H. A population-based study on the association between educational length, prostate-specific antigen testing and use of prostate biopsies. Scand J Urol 2016;50(2):104–9.

19. Protocol Addendum STHLM3MR Main Study

Introduction

This document describes clarifications and adjustments of the study protocol made after its finalization and publication (Protocol version Nordström et al. BMJ Open 2019). All listed adjustments have been approved in consensus by the trial steering committee.

Table of additions and clarifications

Date	Type	Description	Ref Protocol
2019-11-13	Sample size calculation	Decided to increase study size. Power calculation based on assumtions: participation rate (DSMB2; 22%), 13% men randomized; 20% men not following protocol; 55% MRI-negative; size increased to enable secondary contrast comparison s3M+MRI/TBx vs PSA+MRI/TBx (Additional Hypotheses 4). See SAP for details.	13.9
2020-11-27	Clarification	Clarification of wording in hypothesis descriptions.	9
20200108	Definition, inclusion	Men creating referral before 2020-01-09 are included in main analysis.	13.2
20200108	Definition, inclusion	Age is defined as age at referral creation. Men aged 50-74 are included in main analysis	13.2
20200108	Definition, intervention	Men with PSA>40 ng/ml are directly transferred for prioritized clinical workup at Capio St Görans Hospital. Intervention are at the discretion of the clinician, possibly including MRI.	13.4

		Approximately 5/10000 tested men have PSA ≥40 ng/ml	
20200108	Definition, analysis PP	PP (per protocol) analysis: Include systematic pathology report for men in standard arm. Include systematic+target report (PIRADS≥3) for men in experimental arm. Men without systematic bx report are protocol violators and not included in PP. Men in PP must have PSA and Stockholm3-result, otherwise excluded.	13.4
20200115	Definition, inclusion	Invited are defined as men that had letter sent subtracted by letters that were returned	13.10
20200115	Definition, intervention	Complete **pathological report** is defined as containing cancer/benign AND Global Gleason. For men in the experimental arm, this should hold for both pathological reports (TBx/SBx). For men with a “negative MRI” but STHLM3>25% this should hold for SBx pathological report.	13.4
20200115	Definition, intervention	**Global Gleason Score** is defined as the highest of Systematic and Targeted Bx global scores	13.4
20200115	Definition, intervention	Complete MRI is defined as existing MRI report including PIRADS score.	13.4

		Complete MRI is mandatory for inclusion of experimental arm men in the PP.	
20200115	Definition, Study population	Study population is defined as participating men. See further in Statistical Analysis Plan	13.4
2020-01-29	Definition, Study population	Participating men are defined as men with registered consent and blood sample.	13.4

STHLM3 MR Phase II

Randomized diagnostic trial

STATISTICAL ANALYSIS PLAN

Authors

Tobias Nordström[a,b]
Andrea Discacciati[a]
Martin Eklund[a]

[a] Department of Medical Epidemiology and Biostatistics, Karolinska Institutet, Stockholm, Sweden;
[b] Department of Clinical Sciences at Danderyd Hospital, Karolinska Institutet, Stockholm, Sweden.

Revisions

Date	Authors	Reason	Main changes
180217	ME	First draft of SAP	
180515	ME	Feedback after initial DSMB meeting	Hypotheses and aims clarified.
191115	AD	Updated sample size calculations.	Updated sample size calculations to support decision to expand study in order to have sufficient power for within-arm comparisons of Stockholm3 vs. PSA in the experimental arm.
191127	ME/AD/TN	Publication plan	Adjustment definition TBx. Introducing publication plan with 2 main manuscripts. Clarification and specification of the wording of the hypotheses. Clarification of follow-up time.
200324	ME	Covid-19	Allow for potential analysis of the trial in two phases due to the Covid19 pandemic.

201223	ME	Description of post-hoc analyses	Description of analyses where biopsies and biopsy results on men with negative MRI but Stockholm≥25% are ignored.

Contents

Authors 1

Revisions 1

Contents 2

Abbreviations 4

Preface 5

Design 5

Hypotheses 6

Overarching primary hypothesis 6

Additional hypotheses 7

Publications strategy of main study results 7

Publication 1 8

Publication 2 8

Aims and endpoints 9

Primary aim 9

Key secondary aims 9

Additional aims 9

Main endpoints 10

Additional endpoints 10

Study variables 10

Primary endpoints 10

Key secondary endpoints 10

Other secondary endpoints 10

Independent variabels 11

Statistical analysis 12

Study populations 12

Patients’ characteristics 13
Analyses 13
Data structure 13
Contrasts between study arms (unpaired design) 14
Interpretation as relative sensitivity 15
Contrasts within study arm (paired design) 15
Non-inferiority and superiority tests 15
Primary endpoints 16
Key secondary endpoints 16
Secondary endpoints 16
Sample size calculations 16
Original sample size calculation (performed in March 2017) 16
Basic data and assumptions used in the sample size calculations 16
Primary contrast 16
Secondary contrast **Error! Bookmark not defined.**
Updated sample size calculations (performed during Spring and Summer 2019) 17
Subgroup analyses 18
Additional analyses 18
Data Safety Management Board (DSMB) 19
Handling of missing data 19
Covid-19 addendum 200223 19
Appendix 1 20
References 21

Abbreviations

PSA	Prostate specific antigen
S3M	Stockholm3 model
SBx	Systematic biopsy
TBx	Targeted biopsy
TPF	True positive fraction
FPF	False positive fraction
DP	Detection Probability
rTPF	Relative true positive fraction
rFPF	Relative false positive fraction
rDP	Relative Detection Probability
BR	Biopsy rate
rBR	Relative biopsy rate
ITT	Intention-to-treat
PP	Per protocol
MP	Multiple imputation
ICER	Incremental cost-effectiveness ratio
MRI	Magnetic resonance imaging
DSMB	Data safety and monitoring committee
SAP	Statistical analysis plan
PI-RADS	Prostate imaging reporting and data system
ISUP	International Society of Urological Pathology
DRE	Digital rectal examination
SEK	Swedish Kronor
QALY	Quality-adjusted life-years

Preface

This statistical analysis plan (SAP) describes the planned analyses for STHLM3MR Phase 2 (NCT03377881) (herein referred to as STHLM3MR for short). STHLM3MR is a study with first a paired design step for the blood-tests PSA and Stockholm3 and a second randomized step where men with increased risk in the first step (based on either PSA or Stockholm3) are randomized to systematic or MRI+systematic+targeted biopsies, respectively (Figure 1). The planned analyses identified in this SAP will be included in future manuscripts. Exploratory analyses not necessarily identified in this SAP may be performed to support planned analyses. Any post-hoc exploratory or unplanned analyses not specified in this SAP will be identified as such in manuscripts for publication, and added as addenda to the SAP. To ensure blinding, arm allocations are stored in a separate location accessible only by an unblinded statistician. The SAP may be updated during the course of the trial but will be finalized before database lock or any comparative analyses.

Design

The study design is outlined in detail in the study protocol. Figure 1 shows a schematic overview of the study design. The design combines a paired step (PSA vs. Stockholm3) and a randomized step (systematic biopsies vs. MRI plus targeted and systematic biopsies on MRI positive men). The rationale behind the paired first step is that it maximizes statistical power (for a given sample size) without any clear risk of introducing biased comparisons[1]. The different biopsy strategies could also be compared in a paired design (like in e.g. Ahdoot et al.[2], Rouvière et al.[3], and Grönberg et al.[4]), however this markedly increases risk of introducing bias (since bleeding artefacts would interfere with performing the targeted biopsies if the systematic biopsies are performed first, and vice versa, even in the case of different urologists performing the two biopsy techniques within the same man, like in Rouvière et al.[3]). We therefore chose to have a randomized second step to enable higher quality in the comparison of biopsy strategies (systematic vs. MRI plus targeted and systematic biopsies for MRI positive men, defined as PIRADS ≥ 3). For safety reasons, all men with very high risk (Stockholm3 test ≥25%) will be recommended systematic biopsies. It should be noted that we by the Stockholm3 test in this study refer to the Stockholm3 test as described in Ström et al.[5] ***without*** the inclusion of prostate volume and digital rectal examination (DRE) as predictors (i.e., the set of predictors include age, first-degree family history of prostate cancer [yes/no], and previous biopsy [yes/no], total PSA, free PSA, ratio of free/total PSA, hK2, MIC1, MSMB, and a genetic score).

The design permits a large number of comparative contrasts to be performed (constructed of combinations of using either PSA or Stockholm3, using MRI or not, and using targeted biopsies or systematic biopsies or both can be compared). Specifically, the following diagnostic strategies can be compared:

1. PSA+SBx
2. PSA+MRI+TBx
3. PSA+MRI+TBx+SBx
4. S3M+SBx
5. S3M+MRI+TBx
6. S3M+MRI+TBx+SBx

In addition, the following diagnostic strategies are also possible:

7. (PSA | S3M)+SBx
8. (PSA | S3M)+MRI+TBx

9. (PSA | S3M)+MRI+TBx+SBx,

where (PSA | S3M) denotes positive on either PSA or S3M screening tests, SBx denotes systematic biopsies, and TBx denotes targeted biopsy. To be clear, only MRI positive men (PIRADS ≥ 3) are biopsied in the strategies that include MRI (apart from men with S3M ≥ 25%). This is true irrespectively of the biopsy procedure (i.e. SBx is also only performed in MRI positive men in these strategies) (Figure 1). ***The above strategies will in the rest of this document be referred to as Strategy 1, 2, 3, 4, 5, 6, 7, 8, and 9, respectively.***

Randomization stratified on disease risk [sextiles of the Stockholm3 test] will be used. Further, block allocation will be performed, with each block consisting of five men, two of which will be randomized to systematic biopsies and three will be randomized to the MRI arm. This means that randomization guarantees a proportional number of men in each arm and with more evenly distributed characteristics in terms of disease risk and test concordance.

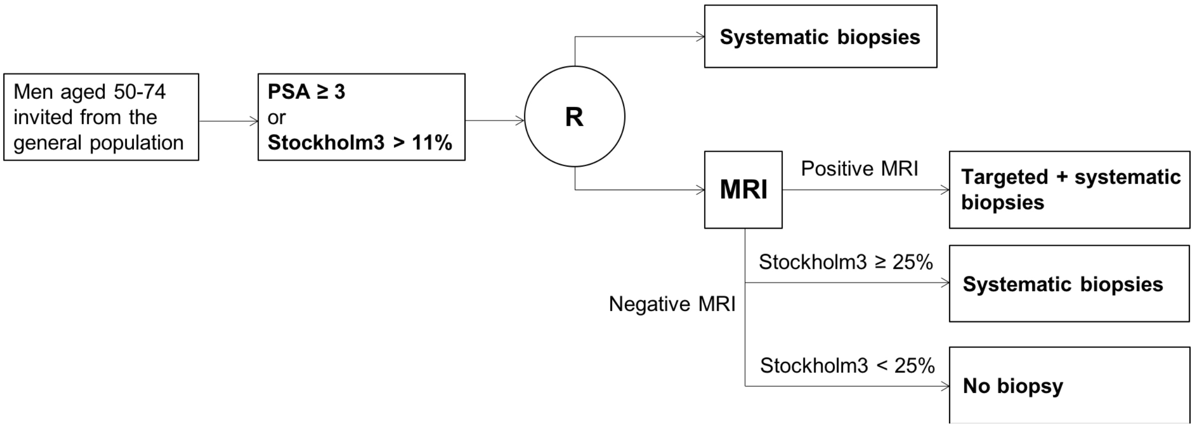

Figure 1: *Overview of the STHLM3 MR Phase 2 study design. Men aged 50-74 are invited to the study from the general population. Blood is sampled from study participants and PSA as well as Stockholm3 are measured. Men with elevated prostate cancer risk (PSA ≥ 3 ng/ml or Stockholm3 ≥ 11%) are randomized to be referred to either systematic biopsies (control arm) or undergo MRI and targeted plus systematic biopsies in case the MRI indicates areas of the prostate suggestive of prostate cancer (PIRADS ≥ 3). The design thus combines a paired step where PSA and Stockholm3 can be compared (paired screen positive design) and a randomized step where systematic biopsies can be contrasted to MRI and subsequent targeted and systematic biopsies for MRI positive men.*

Hypotheses

Overarching primary hypothesis

The overarching primary hypothesis of the STHLM3MRI trial is that a diagnostic pathway using the Stockholm3 test to select men for further workup using MRI followed by targeted biopsies and systematic biopsies in MRI positive men (S3M+MRI+TBx+SBx; Strategy 6) has non-inferior sensitivity for detecting clinically significant cancer (ISUP grade ≥2) and shows superior specificity (reduction in number of performed biopsy procedures and detected ISUP 1 tumours) compared with the diagnostic pathway using systematic biopsies in men with PSA ≥ 3 ng/mL (PSA+SBx; Strategy 1).

Additional hypotheses

1. When compared with performing systematic biopsies for men with elevated risk of prostate cancer in prostate cancer screening, targeted and systematic prostate biopsies performed on MRI positive men has non-inferior sensitivity for detecting clinically significant cancer (ISUP grade group ≥2) and reduces the number of performed biopsy procedures, which also translates to lower proportion of men with elevated risk who experience severe post-biopsy infections. Elevated risk can here be defined using PSA or S3M – we clarify the exact contrasts for testing this hypothesis below.
2. When compared with performing systematic biopsies for men with elevated risk of prostate cancer in prostate cancer screening, targeted biopsies only performed on MRI positive men has non-inferior sensitivity for detecting clinically significant cancer (ISUP grade group ≥2) and reduces the number of performed biopsy procedures, which also translates to lower proportion of men with elevated risk who experience severe post-biopsy infections. Elevated risk can here be defined using PSA or S3M – we will clarify the exact contrasts for testing this hypothesis below.
3. A diagnostic chain consisting of Stockholm3 followed by MRI and targeted+systematic biopsies (S3M+MRI+TBx+SBx) versus a diagnostic chain based on PSA ≥3 ng/ml followed by MRI and targeted+systematic biopsies (PSA+MRI+TBx+SBx) will lead to: non-inferior sensitivity for detecting clinically significant cancer (ISUP grade group ≥ 2); an inferior sensitivity for ISUP1 cancers (i.e. reduced overdiagnosis); and a reduction in the number of MRI examinations and performed biopsies.
4. A diagnostic pathway using the Stockholm3 test to select men for further workup using MRI followed by ONLY targeted biopsies (S3M+MRI+TBx) has non-inferior sensitivity for detecting clinically significant cancer (ISUP grade group ≥2) and reduces the number of performed biopsy procedures compared with a diagnostic pathway using systematic biopsies in men with PSA ≥3 ng/mL (PSA+SBx).
5. Biopsy compliance is higher after biopsy is recommended based on MRI compared to recommended without MRI.
6. SBx in the MRI arm has superior sensitivity than SBx in the non-MRI arm (due to cognitive fusion).
7. A diagnostic chain consisting of Stockholm3 followed by MRI and targeted+systematic biopsies (S3M+MRI+TBx+SBx) is cost-effective (ICER < 750 000 SEK per QALY gained) compared to a diagnostic chain based on PSA ≥3 ng/ml followed by MRI and targeted+systematic biopsies (PSA+MRI+TBx+SBx) due to reductions in number of performed procedures (men undergoing MRI and biopsy).
8. A diagnostic chain using the Stockholm3 test to select men for further workup using MRI and targeted+systematic biopsies (S3M+MRI+TBx+SBx) is cost-effective compared to a diagnostic chain using systematic biopsies in men with PSA ≥3 ng/ml (PSA+SBx).
9. Adding prostate volume as a variable in the diagnostic chain with Stockholm3 test (i.e. using the full Stockholm3 model described in Ström et al.[5]) and MRI/Fusion biopsies improves model precision, leading to further improvements in specificity compared to the use of the Stockholm3 test without the inclusion of prostate volume.

Publications strategy of main study results

A large number of publications will likely be written based on the data collected within the STHLM3MRI trial. The main study results from the trial will be reported in the two first publications:

Publication 1

Publication 1 will as primary contrast report the comparison of Strategy 3 vs. 1 (PSA+MRI+TBx+SBx vs PSA+SBx). Additional analysis in the report will include contrasts of Strategies 2 vs 1.

This comparison uses only the randomized step of the trial design and is motivated by the need to provide level 1 evidence about the performance of MRI+targeted+systematic biopsies versus systematic biopsies alone in men with PSA ≥3 ng/ml in a screening-by-invitation context, where such data is lacking entirely. The choice of using systematic biopsies without MRI as the comparator hinges on the fact that, presently, this is the typical diagnostic strategy offered to men with an elevated PSA (Ahmed et al.[6]). Furthermore, level 1 evidence about a mortality benefit from early detection of prostate cancer is available only for a diagnostic strategy based on PSA+systematic biopsies (Schröder et al. 2009[7] and 2014[8]). Thus, Publication 1 will assess whether introducing PSA+MRI+TBx+SB into prostate cancer screening can diagnose clinically significant prostate cancer with non-inferior sensitivity to PSA+SB, for which there is level 1 evidence of a reduction in prostate cancer mortality. Publication 1 will cover additional hypotheses 1 and 2.

Publication 2

Publication 2 will report the contrasts of Strategy 6 vs. 3 (S3M+TBx+SBx vs. PSA+TBx+SBx) and Strategy 6 vs. 1 (S3M+TBx+SBx vs. PSA+SBx), where the latter contrast corresponds to assessing the performance of the entire diagnostic chain of using Stockholm3 test to select men for further workup using MRI followed by targeted biopsies and systematic biopsies compared to using PSA followed by systematic biopsies (i.e. testing the overarching primary hypothesis of the trial). Sensitivity analyses will include contrast of Strategy 5 vs. 2 and Strategy 5 vs. 1. In addition, for the contrast of Strategy 6 vs. 1, we will – if Stockholm3 ≥ 11% is more sensitive than PSA ≥ 3 – also report results at the operating point of Stockholm3 (i.e. the Stockholm3 cutoff) that gives equal sensitivity as PSA ≥ 3 within the experimental arm (analogously to how we performed the STHLM3 trial, see Grönberg et al.[9]), see the “Additional analysis” section in this document for more information.

This comparison is motivated by the fact that MRI+targeted biopsy will be used more and more frequently, with the possibility of them eventually replacing systematic biopsies as the de-facto standard diagnostic tool[1]. For example, the National Institute for Health Care and Excellence (NICE) in the UK already recommends MRI as “the first-line investigation for people with suspected clinically localised prostate cancer”[2]. Therefore, we aim to compare the Stockholm3 test with the PSA test – in terms of sensitivity (ISUP≥2), specificity (ISUP1), number of biopsies and number of MRI scans – as a tool to select men for further workup, as well as compare the entire diagnostic chain of using Stockholm3 followed by MRI and TBx+SBx to the traditional diagnostic chain of using PSA followed by SBx (for which there exists level 1 evidence of reduced prostate cancer specific mortality when used for prostate cancer screening (Schröder et al. 2009[7] and 2014[8])). Publication 2 will cover the primary overarching hypothesis, as well as additional hypotheses 3 and 4.

Another way to motivate the order of these two publications is that the first publication will assess whether MRI and TBx+SBx improves diagnostic accuracy in a population based

---

[1] As also pointed out in by Professor Mark Emberton and Professor Caroline M. Moore in the review of the study protocol [Nordström et al, BMJ Open 2019]. The comments are now available at the journal’s website.

[2] See https://www.nice.org.uk/guidance/NG131/chapter/recommendations#multiparametric-mri-and-protocol-for-active-surveillance

screening-by-invitation setting, as it seems to do in clinical cohorts (Kasivisvanathan et al.[10] and Ahdoot et al.[2], Rouvière et al.[3], and Grönberg et al.[4]), whereas the second publication will assess whether Stockholm3 can improve selection of men to undergo MRI (an key point since MRI is an expensive and scarce resource and population-based screening involving MRI will lead to large number of MRI examinations).

The results presented in Publications 1 and 2 will thus together cover the testing of the overarching primary hypothesis, as well as additional hypothesis 1, 2, 3, 4, and 5.

Aims and endpoints

The primary aim and key secondary aims of this trial are described below (corresponding with the overarching primary hypothesis and additional hypothesis 1-4 above), together with definitions of study variables (independent variables and outcome variables). A description of how and on which data statistical testing will be performed is specified in the Statistical Analysis section.

Primary aim

To test whether a diagnostic pathway using the Stockholm3 test to select men for further workup using MRI followed by targeted biopsies and systematic biopsies (Strategy 6; S3M+MRI+TBx+SBx) has non-inferior sensitivity for detecting clinically significant cancer (ISUP grade ≥2) and shows superior specificity (reduction in number of performed biopsy procedures and detected ISUP 1 tumours) compared with the diagnostic pathway using systematic biopsies in men with PSA ≥ 3 ng/mL (Strategy 1; PSA+SBx).

Key secondary aims

1. To test whether targeted prostate and systematic biopsies performed in MRI positive men will lead to: non-inferior sensitivity for detecting clinically significant cancer (ISUP grade group ≥2); reduced number of performed biopsy procedures; and lower proportion of men with elevated risk who experience severe post-biopsy infections compared to systematic biopsies for men with elevated risk (defined as being positive on PSA and/or Stockholm3) of prostate cancer in prostate cancer screening.
2. To test whether targeted prostate biopsies performed in MRI positive men will lead to: non-inferior sensitivity for detecting clinically significant cancer (ISUP grade ≥2); reduced number of performed biopsy procedures; and lower proportion of men with elevated risk who experience severe post-biopsy infections compared to systematic biopsies for men with elevated risk (defined as being positive on PSA and/or Stockholm3) of prostate cancer in prostate cancer screening.
3. To test whether a diagnostic chain consisting of Stockholm3 followed by MRI and targeted+systematic biopsies versus a diagnostic chain based on PSA ≥3 ng/ml followed by MRI and targeted+systematic biopsies will lead to: non-inferior sensitivity for detecting clinically significant cancer (ISUP grade group ≥ 2); an inferior sensitivity for ISUP1 cancers (i.e. reduced overdiagnosis); and a reduction in the number of MRI examinations and performed biopsies.
4. To test whether a diagnostic pathway using the Stockholm3 test to select men for further workup using MRI followed by ONLY targeted biopsies (S3M+MRI+TBx) has non-inferior sensitivity for detecting clinically significant cancer (ISUP grade ≥2) and reduces the number of performed biopsy procedures compared with a diagnostic pathway using systematic biopsies in men with PSA ≥3 ng/mL (PSA+SBx).

Additional aims

Additional aims corresponding to hypotheses 4-8 above will be assessed.

Main endpoints

Primary endpoint:

1. Diagnosed ISUP grade ≥ 2 cancers

Key secondary endpoints

2. Diagnosed ISUP grade 1 cancers
3. Performed biopsies
4. Performed MRI examinations

Additional endpoints

The primary and secondary endpoints are reported in the tables below. All these endpoints can be used for comparisons between the nine diagnostic strategies listed in the 'Design' section.

Study variables

Primary endpoints

Variable	Measure	Comment
Clinically significant prostate cancer	Yes/No	ISUP ≥ 2

Key secondary endpoints

Variable	Measure	Comment
Non-clinically significant prostate cancer	Yes/No	ISUP = 1
Biopsy performed	Yes/No	Any biopsy procedure performed
MRI performed	Yes/No	MR procedure performed

Other secondary endpoints

Variable	Measure	Comment
Any cancerous finding	Yes/No	ISUP ≥ 1
ISUP ≥ 3 prostate cancer	Yes/No	ISUP ≥ 3
ISUP 2 through 5	Yes/No	Four separate endpoints:

		(1) ISUP=2, (2) ISUP=3, (3) ISUP=4, and (4) ISUP=5
Lesion volume	ml	Ellipsoid calc (4*pi*h*w*l /3)(4*pi*h*w*l /3)<br>Total volume of all reported lesions PIRADS≥3
Number of biopsies	Integer	No of reported biopsy needles
Cancer length	mm	total mm of cancer in reported biopsy
% Gleason ≥4	%	summarized % Gleason ≥4 in needles with cancer
Maximum cancer core length	mm	maximum length of cancer in any core
Maximum GS ≥ 4 cancer core length	mm	maximum length of Gleason ≥ 4 cancer in any core
Serious adverse events	Yes/No	(1) Hospitalisation within 30 days after biopsy procedure, (2) infection treated with antibiotics within 30 days after biopsy procedure, or (3) death within 30 days after biopsy procedure

Independent variables

Variable	Measure	Comment
Age	Years	At referral creation
Previous prostate biopsy	Yes/No	Patient self-reported
Family history of prostate cancer	Yes/No	Any first degree relative with prostate cancer
PSA	ng/ml	At blood test
free PSA	ng/ml	At blood test

Stockholm3 risk score	% risk of ISUP≥2	At blood test
prostate volume	ml	MRI defined
PI-RADS	1–5 (integer)	Maximum PI-RADS score

Statistical analysis

All analyses will be performed after the study is completed and the database is released. All statistics, including tables, figures and listings, will be performed using R version >3.5. Men creating referral before 2020-01-09 are included in main analysis. All participants with will be followed a minimum of 200 days after receiving the result from their blood sample (PSA and Stockholm3 score). In addition, biopsied men will be followed at least 30 days post-biopsy to monitor adverse events, and participants undergoing radical prostatectomy prior to database lock will be followed until pathology results from the prostatectomy are available.

Study populations

1. *ITT population* includes all men who:
    a. signed the written informed consent to participate in the study,
    b. fulfilled all inclusion criteria and none of the exclusion criteria,
    c. were randomised to either study arm.

Conditions for excluding patients from the ITT population, based on deviations from the study protocol:

**Randomisation arm**	**MRI**	**No biopsy**	**SBx only**	**TBx only**	**SBx and TBx**
Standard arm	MRI or no MRI	Include	Include	Include	Include
Experimental arm	MRI or no MRI	Include	Include	Include	Include

Randomised allocation and analysis group for ITT analyses:

**Randomisation arm**	**Test received**	**Analysis group**
Standard arm	Systematic biopsy (with or without MRI)	Standard arm
Standard arm	Targeted biopsy	Standard arm
Standard arm	Systematic biopsy and Targeted biopsy	Standard arm
Standard arm	No biopsy / Other	Standard arm
Experimental arm	Systematic biopsy (with or without MRI)	Experimental arm
Experimental arm	Targeted biopsy	Experimental arm

Experimental arm	Systematic biopsy and Targeted biopsy	Experimental arm
Experimental arm	No biopsy / Other	Experimental arm

2. *PP population* includes men who:
    a. are included in the ITT population,
    b. have a valid PSA value and Stockholm3 score,
    c. have a complete systematic pathology report if randomised to standard arm (but no targeted pathology report),
    d. have a complete MRI report if randomised to experimental arm and (i), (ii), or (iii), as appropriate,
        i. have a complete systematic and a complete targeted pathology report if PIRADS≥3,
        ii. have a complete systematic pathology report if PIRADS<3 and Stockholm3≥25% (but no targeted pathology report),
        iii. did not undergo any biopsy if PIRADS<3 and Stockholm3<25%.

Conditions for excluding patients from the PP population, based on deviations from the study protocol:

Randomisation arm	Further specifications	No biopsy	SBx only	TBx only	SBx and TBx
Standard arm	No MRI	Exclude	Include	Exclude	Exclude
Standard arm	MRI	Exclude	Exclude	Exclude	Exclude
Experimental arm	No MRI	Exclude	Exclude	Exclude	Exclude
Experimental arm	MRI PIRADS≥3	Exclude	Exclude	Exclude	Include
Experimental arm	MRI PIRADS<3 and Stockholm3≥25%	Exclude	Include	Exclude	Exclude
Experimental arm	MRI PIRADS<3 and Stockholm3<25%	Include	Exclude	Exclude	Exclude

The analyses will be performed and reported on both the ITT and PP population.

Patients' characteristics

Patients' characteristics will be presented with descriptive statistics, overall, by study arm, and/or by screening test (positive/negative), as appropriate. Continuous variables will be summarized using measures of central tendency and variability. Categorical variables will be summarized using absolute and relative frequencies. No formal statistical testing will be performed.

Analyses

Data structure

The table below lays out the general data structure for the STHLM3-MRI trial.

**Standard arm**

	Endpoint = positive (yes)		Endpoint = negative (no)	
	PSA test positive	PSA test negative	PSA test positive	PSA test negative
Stockholm3 test positive	a	b	e	f
Stockholm3 test negative	c	[d]	g	[h]
**Experimental arm**				
	Endpoint = positive (yes)		Endpoint = negative (no)	
	PSA test positive	PSA test negative	PSA test positive	PSA test negative
Stockholm3 test positive	a’	b’	e’	f’
Stockholm3 test negative	c’	[d’]	g’	[h’]

Note: since the study protocol dictates that only those patients who screen positive on either screening test are referred for further work-up, the number of patients reported between brackets are unknown. In a standard screen-positive study, the total number of patients [d]+[h] (and [d’]+[h’]) is known. However, this study combines a paired and a randomised design and only those men who screen positive on either test are randomised. Therefore, the quantities [d]+[h] and [d’]+[h’] are unknown in the present study.

Contrasts between study arms (unpaired design)

Analyses will compare the difference in detection probabilities (DPs) between study arms. The DP is the probability of being endpoint-positive given the study arm and, possibly, either or both screening tests being positive. For example the DP of ISUP1 in men randomised to the experimental arm and Stockholm3-positive is equal to Pr(ISUP1 | Stockholm3≥11%, Experimental arm).

Absolute scale. The absolute difference in DPs is defined as the DP in the experimental arm minus the DP in the standard arm ($\Delta DP = DP_{Exp} - DP_{Std}$) or vice versa, as appropriate. It is estimated by plugging into the formula the observed proportions. An approximate 100(1-α)% two-sided Wald confidence interval for ΔDP is calculated as

$$\widehat{\Delta DP} \pm z_{\alpha/2}\sqrt{\frac{\widehat{DP}_{Exp}*(1-\widehat{DP}_{Exp})}{n_{Exp}} + \frac{\widehat{DP}_{Std}*(1-\widehat{DP}_{Std})}{n_{Std}}}.$$

Relative scale. The relative difference in DPs is defined as the DP in the experimental arm divided by the DP in the standard arm ($rDP = DP_{Exp}/DP_{Std}$) or vice versa, as appropriate. It is estimated by plugging into the formula the observed proportions. An approximate 100(1-α)% two-sided Wald confidence interval for rDP is calculated as

$$exp\left(log(\widehat{rDP}) \pm z_{\alpha/2}\sqrt{\frac{1}{\widehat{DP}_{Exp}*n_{Exp}} - \frac{1}{n_{Exp}} + \frac{1}{\widehat{DP}_{Std}*n_{Std}} - \frac{1}{n_{Std}}}\right).$$

Contrasts 3 vs 1, 2 vs 1, and 9 vs 7 will be analysed using absolute differences (Study 1). Contrasts 6 vs 1, and 5 vs 1 will be analysed using relative differences (Study 2).

Interpretation as relative sensitivity

It should be noted that rDPs can (under the assumption of no false positive biopsies, see SAP Appendix 1) be interpreted as relative true positive fractions (rTPF) (ie, relative sensitivities).

Contrasts within study arm (paired design)

Analyses will compare the true positive fraction between Stockholm3 and PSA, within either study arm. Comparisons will be made on a relative scale. rTPF is defined as $TPF_{Stockholm3}/TPF_{PSA}$ or vice versa, as appropriate.

The rTPF (standard arm) is estimated as (a+b)/(a+c) (or (a+c)/(a+b), as appropriate) and an approximate 100(1-α)% two-sided confidence interval for rTPF is calculated as

$$exp\left(log(\widehat{rTPF}) \pm z_{\alpha/2}\sqrt{\frac{b+c}{(a+b)(a+c)}}\right).$$

Analogous formulas are used for comparisons within the experimental arm and for the relative False Positive Fraction.

Note: the quantity (a+b)/(a+c) estimates the rTPF in both the enrolled and randomised population. It also estimates the ratio of detection probabilities like Pr(ISUP≥2, Stockholm≥11% | Experimental arm)/Pr(ISUP≥2, PSA≥3 ng/ml | Experimental arm).

Non-inferiority and superiority tests

The null and the alternative hypothesis for a non-inferiority test for the ΔDP are:

$$H_0: \Delta DP \leq -\delta$$
$$H_a: \Delta DP > -\delta$$

where $\delta > 0$ is the non-inferiority margin. This means that non-inferiority for a specific endpoint will be claimed if the lower boundary of the two-sided $(2\alpha \times 100)\%$ confidence interval for the ΔDP does not cover $-\delta$.

The null and the alternative hypothesis for a superiority test for the ΔDP are:

$$H_0: \Delta DP \leq \theta$$
$$H_a: \Delta DP > \theta$$

where $\theta \geq 0$ is the superiority margin. This means that superiority for a specific endpoint will be claimed if the lower boundary of the two-sided $(2\alpha \times 100)\%$ confidence interval for the ΔDP does not cover θ.

For comparisons on a relative scale (rTPF), the null and the alternative hypothesis for non-inferiority and superiority tests are

$$H_0: rDP \leq exp(-\delta)$$

$$H_a: rDP > exp(-\delta)$$

and

$$H_0: rDP \leq exp(\theta)$$
$$H_a: rDP > exp(\theta)$$

respectively, with non-inferiority and superiority margins equal to $\delta > 0$ and $\theta \geq 0$.

One-sided p-values will be calculated based on the test considered (non-inferiority or superiority).

Switching from non-inferiority to superiority: if the two-sided $(2(1-\alpha) \times 100)\%$ confidence interval for ΔDP (rTPF) not only lies entirely above the non-inferiority margin, but also above the superiority margin, superiority will be claimed at the same alpha-level set for the non-inferiority test. In this case, we will also calculate the p-value associated with a test for superiority.

Primary endpoints

Contrasts between study arms: we will assess the non-inferiority of the experimental arm versus the standard arm in detecting ISUP≥2 cancers ($\delta = 0.04$ or $exp(-\delta) = 0.78$, as appropriate). The α level is set to 0.025. Two-sided 95% confidence intervals will be reported.

Contrasts within study arms: we will assess the non-inferiority of the Stockholm3 test versus PSA in detecting ISUP≥2 cancers ($exp(-\delta) = 0.78$) and the superiority of PSA versus the Stockholm3 test in detecting ISUP1 cancers (i.e. a lower proportion of ISUP1 cancer detected according to the Stockholm3 test) ($exp(-\theta) = 1$). The α level is set to 0.025 for both tests. Two-sided 95% confidence intervals will be reported.

Key secondary endpoints

Contrasts between study arms: we will assess the superiority of the standard arm versus the experimental arm in detecting ISUP1 cancers (i.e. a lower proportion of ISUP1 cancer detected in the experimental arm) ($\theta = 0$ or $exp(-\theta) = 1$, as appropriate). The α level is set to 0.025. Two-sided 95% confidence intervals will be reported.

Contrasts within study arms: we will assess the superiority of PSA versus the Stockholm3 test in detecting ISUP1 cancers (i.e. a lower proportion of ISUP1 cancer detected according to the Stockholm3 test) ($exp(-\theta) = 1$). The α level is set to 0.025. Two-sided 95% confidence intervals will be reported.

Secondary endpoints

We will report the proportion of men with post-biopsy SAEs (see table "Secondary endpoints") by study arm, where applicable.

Sample size calculations

Original sample size calculation (performed in March 2017)

Basic data and assumptions used in the sample size calculations

We used data from the STHLM3 trial[10] for sample size calculations. In this data, 18% of men with PSA ≥ 3 had a clinically significant prostate cancer when biopsied with SBx. We further

noted that rTPF=1.25 for clinically significant prostate cancer comparing MRI+TBx with SBx based on the results from a meta-analysis (Schoots et al. 2015[3]). We set the noninferiority delta to 4 percentage points for demonstrating noninferiority with respect to sensitivity of clinically significant prostate cancer. We set the alpha to 0.025.

Primary contrast

This study was originally powered for the contrast of Strategy 6 vs 1. Simulating 1000 trials (by bootstrapping from the STHLM3 data) under the assumptions outlined in the preceding section 303 men need to biopsied in the SBx arm based on PSA ≥ 3 to have 80% power to demonstrate non-inferior sensitivity of S3M+MRI+TBx compared with PSA+SBx. This means that at least 415 men need to be biopsied in the SBx arm (since some men are not randomized based on PSA ≥ 3 but on S3M ≥ 11%) and, consequently, 623 to the MRI arm (because of the 2:3 randomization). Total number of men undergoing workup according to protocol (SBx in the no MRI arm and MRI and TBx if PI-RADS ≥ 3 in the MRI arm) is thus 1038. Assuming 20% dropout, 1300 men need to be randomized.

Updated sample size calculations (performed during Spring and Summer 2019)

We revised the sample size calculations above in order to have sufficient statistical power to answer comparisons of Stockholm3 vs. PSA within the experimental arm.

The updated, final sample size is the result of a balance between time and financial constraints on one hand and the need to maximise the power for contrasting Strategies 6 vs. 3 on the other hand. In fact, different assumptions about the joint probability of ISUP≥2 and screening positive on the PSA test [Pr(ISUP≥2, PSA≥3 ng/ml)] and about the TPF of the PSA test [Pr(PSA≥3 ng/ml | ISUP≥2)] lead to different required sample sizes.

Based on the sample size calculation, we decided to increase the number of men invited into the study to ~50000. Based on an updated estimate of the expected participation rate (25%), this will lead to ~12500 men included in the study and to ~2100 randomised men based on PSA or Stockholm3 (assuming ~16.5% of the enrolled men will test positive on either screening test; 13% passed on PSA alone, leading to ~1600 randomized men with PSA ≥ 3 ng/ml), ~1700 of whom will complete the diagnostic chain, assuming a 20% drop-out rate.

The updated sample size will give:

1. 50–90% power for the contrast S3M+TBx+SBx versus PSA+TBx+SBx. This is based on the following assumptions: 0.015–0.03 probability of detecting ISUP≥2 and screening positive on the PSA test (Grönberg et al. 2015[9]), 0.57–0.63 TPF for the PSA test (Thompson et al. 2005), rTPF (Stockholm3≥11% vs PSA≥3) equal to 1 (Grönberg et al. 2015[9]), and a conservative DDR estimate (Alonzo, Pepe, and Moskowitz 2002[13]). The non-inferiority margin was set to $\delta = -log(0.78)$ and alpha to 0.025.

   Note: the contrast S3M+TBx+SBx versus PSA+TBx+SBx is nested within the experimental study arm, where about 7500 men will be included (due to the 2:3 randomisation). We further assumed a 20% drop-out rate, leading to 6000 men available for the analyses.

DR_psa	TPR_psa	power
0.015	0.57	0.48
0.020	0.57	0.60
0.025	0.57	0.70
0.030	0.57	0.77
0.015	0.59	0.52
0.020	0.59	0.64
0.025	0.59	0.73
0.030	0.59	0.81

0.015	0.61	0.55
0.020	0.61	0.67
0.025	0.61	0.77
0.030	0.61	0.84
0.015	0.63	0.59
0.020	0.63	0.71
0.025	0.63	0.80
0.030	0.63	0.87

2. More than 90% power for the contrast PSA+MRI+TBx+SBx vs PSA+SBx (rDP = 1.3, noninferiority margin for ∆DP $\delta = 0.04$, alpha = 0.025).

Thus, contrast 1 drives the required samples size of the study. We therefore powered the study (with respect to sample size for sending out invitations and enrolling participants) to have sufficient statistical power to answer comparisons of Stockholm3 vs. PSA within the experimental arm.

Note: no correction for multiple comparisons was made. This means that each of the three tests (with respect to the ISUP 2, ISUP 1, and biopsy endpoints) has an approximate type I error rate of 2.5% if the corresponding null hypothesis is true. If all three null hypotheses are true and we assume the tests to be independent, the overall type I error rate is approximately 7%. In reality however, these hypotheses are strongly correlated. Thus, the overall type 1 error rate is bounded below by 2.5% and above by 7%.

R code for the power calculations is available at:
https://gist.github.com/anddis/fc1a265d102b509b0eacd59ab065661a

Subgroup analyses

Subgroup analyses will be performed for the following subpopulations:

- Age: [50, 60), [60, 70), [70, 75) years
- PSA: [1.5, 3)[3], [3, 4), [4,10), [10, +inf) ng/mL
- Screen-naive vs not screen-naive patients
- Biopsy-naive vs not biopsy-naive patients

Statistical tests for effect heterogeneity across subpopulations will be performed by jointly testing the interaction (product) terms in generalised linear models or marginal models (Pepe and Alonzo 2001), as appropriate. No correction for multiple comparisons will be made.

Additional analyses

Due to updates in the Stockholm3 assay system to reduce measurement errors in the biomarkers included in the Stockholm3 risk prediction model, Stockholm3 ≥ 11% as a selection criterion for randomization may be more sensitive than PSA ≥ 3 (i.e. more men with clinically significant prostate cancer will be randomized based on the criterion Stockholm3 ≥ 11% compared with PSA ≥ 3). If this turns out to be true, we will perform analyses where we “count backwards” (increase the S3M cutoff) and compare biopsy rates at identical sensitivity for clinically significant prostate cancer when comparing diagnostic strategies involving S3M compared with PSA ≥ 3 (as described in Grönberg et al.[9]).

We will artificially randomise (2:3) those men who screened negative on both screening tests. By doing this, the totals [d]+[h] and [d’]+[h’] will become known, which in turn will allow the estimation of quantities like Pr(ISUP≥2, Stockholm3≥11% | Standard arm) (i.e., the probability of ISUP≥2 and Stockholm3≥11% in enrolled men randomised to the standard arm). Contrasts between the two study arms with respect to these quantities will be

[3] Where applicable.

performed using the same methodology described in the section “Contrasts between study arms (unpaired design)”.

We may in additional analyses use regression models to model the DP, TPF, and FPF given covariates. We will employ standard generalised linear models or marginal models (Pepe and Alonzo 2001[14]), as appropriate.

Data Safety Management Board (DSMB)

See protocol.

Handling of missing data

Missing data with respect to outcome data (most importantly, participants who are recommended biopsy but never undergo the procedure) will primarily be handled by performing analyses on both the ITT and the PP populations. The analysis on the PP population inherently makes a missing-completely-at-random (MCAR) assumption. However, there is a chance that there is uneven dropout levels in the two arms. For example, men randomized to undergo MRI may to a higher degree choose to undergo biopsy since there is visual feedback of a lesion. If deemed necessary to understand and interpret study results, we may therefore perform multiple imputation based on the Stockholm3 score to impute outcomes for men who drop out of the study before the biopsy is performed. Briefly, if imputation is performed, it will follow the following protocol:

- *Systematic biopsy arm.* The Stockholm3 test, which is calibrated to systematic biopsy outcomes, will be used to impute biopsy outcome on men who did not undergo biopsy (despite a study recommendation to do so) by performing a Bernoulli experiment using the predicted Stockholm3 risk score for ISUP 2 cancer as a parameter. The analysis will be performed on 1000 multiple imputation datasets and summarized.
- *Experimental biopsy arm.* We will, by using data with the STHLM3MRI trial, fit a model to associate Stockholm3 test and PI-RADS score result to TBx outcome (Stockholm3-TBx). Using this model, we will proceed in a similar way as for the control arm. I.e., we will repeatedly impute outcome using Bernoulli experiments with the predictions from the Stockholm3-TBx model as parameter. The analysis will be performed on 1000 multiple imputation datasets and summarized.

Covid-19 addendum 200223

The Covid-19 pandemic puts a tremendous strain on the entire healthcare system, meaning that the STHLM3-MRI phase II study will be impacted and lead times for patients in the trial will become potentially very long. It is not unlikely likely (at the time of writing 200223) that the study will be prolonged by many months and even years. Therefore, we have decided to open up for the possibility to report on endpoints as they mature. I.e., if STHLM3MRI is unable to continue recruit participants and perform tests according to the study protocol, we open for the possibility to not have one finalized database lock that will be used for all analyses. Rather, we may then lock a database for a specific analysis when there is enough data in the study to test the hypothesis corresponding to the analysis. In particular, we already have enough data collected in the trial for Publication 1. From an ethical point of view, we believe this is the least bad possible approach under the current circumstances. We have a large dataset already collected in the study and we believe that it makes sense to use these data to benefit of patients as soon as possible for the endpoints and analyses that are possible to analyse, rather than waiting for a limited set of men who are left in the study and -- due to Covid-19 -- may not be able to complete the study protocol for a very long time. This plan has been communicated to and approved by the trial’s DSMB.

Additional comment written 200905: The trial could be completed despite the covid-19 pandemic, and the addendum above will not have to be activated. This means that we will have one single database lock for performing all analyses, as originally planned.

Post-hoc analyses

Ignoring biopsy results on men with negative MRI and Stockholm3 ≥ 25%

In order to estimate results in the counterfactual scenario where participants with negative MRI and Stockholm3 ≥25% would not have been referred to undergo systematic biopsy, we will perform analyses where these biopsies are ignored. To be clear, we will not exclude these men from the analyses, but ignore their biopsy and biopsy outcome (i.e., they would enter the analyses as not having had a biopsy or any potential cancer diagnosis). The participants with a negative MRI and Stockholm3 ≥25% will in these analyses thus contribute to the counts of performed MRI scans, but not to the count of biopsies or the cancer count. Apart from this, the analyses will be performed identically as detailed above.

Appendix 1

Let T and S be the events "SBx+TBx positive for a specific ISUP grade" (eg, ISUP≥2) and "SBx positive for a specific ISUP grade", respectively. Let P be the event "PSA screening test above 3 ng/ml". Let D be the event "the subject is positive for a specific ISUP grade (true, unobservable status)".

The main between-arm contrast of Study 1, expressed in relative terms, is given by

$\frac{\Pr(T=1|P=1)}{\Pr(S=1|P=1)}$.

This can be rewritten as:

$$\frac{\Pr(T=1|P=1)}{\Pr(S=1|P=1)} = \frac{\Pr(T=1,D=1|P=1)+\Pr(T=1,D=0|P=1)}{\Pr(S=1,D=1|P=1)+\Pr(S=1,D=0|P=1)}$$

$$= \frac{\Pr(T=1,D=1|P=1)+\Pr(T=1|D=0,P=1)\Pr(D=0|P=1)}{\Pr(S=1,D=1|P=1)+\Pr(S=1|D=0,P=1)\Pr(D=0|P=1)} \text{ [assumption]}$$

$$= \frac{\Pr(T=1|D=1,P=1)\Pr(D=1|P=1)}{\Pr(S=1|D=1,P=1)\Pr(D=1|P=1)} = \frac{\Pr(T=1|D=1,P=1)}{\Pr(S=1|D=1,P=1)} = rTPF$$

The third equality holds under the assumption that the FPFs $\Pr(T=1|D=0,P=1)$ and $\Pr(S=1|D=0,P=1)$ are equal to zero, while the fourth equality hinges on the fact that — because of randomisation— the probabilities $Pr(D=1|P=1)$ in the two study arms are the same in expectation.

The equation above can be extended to the other between-arm contrasts.

References

1. Pepe MS. *The Statistical Evaluation of Medical Tests for Classification and Prediction*. 1. publ. in paperback. Oxford Univ. Press; 2004.

2. Ahdoot M, Wilbur AR, Reese SE, et al. MRI-Targeted, Systematic, and Combined Biopsy for Prostate Cancer Diagnosis. *N Engl J Med*. 2020;382(10):917-928. doi:10.1056/NEJMoa1910038

3. Rouvière O, Puech P, Renard-Penna R, et al. Use of prostate systematic and targeted biopsy on the basis of multiparametric MRI in biopsy-naive patients (MRI-FIRST): a prospective, multicentre, paired diagnostic study. *Lancet Oncol*. 2019;20(1):100-109. doi:10.1016/S1470-2045(18)30569-2

4. Grönberg H, Eklund M, Picker W, et al. Prostate Cancer Diagnostics Using a Combination of the Stockholm3 Blood Test and Multiparametric Magnetic Resonance Imaging. *Eur Urol*. 2018;74(6):722-728. doi:10.1016/j.eururo.2018.06.022

5. Ström P, Nordström T, Aly M, Egevad L, Grönberg H, Eklund M. The Stockholm-3 Model for Prostate Cancer Detection: Algorithm Update, Biomarker Contribution, and Reflex Test Potential. *Eur Urol*. 2018;74(2):204-210. doi:10.1016/j.eururo.2017.12.028

6. Ahmed HU, El-Shater Bosaily A, Brown LC, et al. Diagnostic accuracy of multi-parametric MRI and TRUS biopsy in prostate cancer (PROMIS): a paired validating confi rmatory study. *www.thelancet.com*. 2017;389. doi:10.1016/S0140-6736(16)32401-1

7. Schröder FH, Hugosson J, Roobol MJ, et al. Screening and Prostate-Cancer Mortality in a Randomized European Study. *N Engl J Med*. 2009;360(13):1320-1328. doi:10.1056/NEJMoa0810084

8. Schröder FH, Hugosson J, Roobol MJ, et al. Screening and prostate cancer mortality: results of the European Randomised Study of Screening for Prostate Cancer (ERSPC) at 13 years of follow-up. *Lancet Lond Engl*. 2014;384(9959):2027-2035. doi:10.1016/S0140-6736(14)60525-0

9. Grönberg H, Adolfsson J, Aly M, et al. Prostate cancer screening in men aged 50 to 69 years (STHLM3): a prospective population-based diagnostic study. *www.thelancet.com/oncology*. 2015;16. doi:10.1016/S1470-2045(15)00361-7

10. Kasivisvanathan V, Rannikko AS, Borghi M, et al. MRI-Targeted or Standard Biopsy for Prostate-Cancer Diagnosis. *N Engl J Med*. 2018;378(19):1767-1777. doi:10.1056/NEJMoa1801993

11. Schoots IG, Roobol MJ, Nieboer D, Bangma CH, Steyerberg EW, Hunink MGM. Magnetic Resonance Imaging–targeted Biopsy May Enhance the Diagnostic Accuracy of Significant Prostate Cancer Detection Compared to Standard Transrectal Ultrasound-guided Biopsy: A Systematic Review and Meta-analysis. *Eur Urol*. 2015;68(3):438-450. doi:10.1016/j.eururo.2014.11.037

12. Thompson IM. Operating Characteristics of Prostate-Specific Antigen in Men With an Initial PSA Level of 3.0 ng/mL or Lower. *JAMA*. 2005;294(1):66. doi:10.1001/jama.294.1.66

13. Alonzo TA, Pepe MS, Moskowitz CS. Sample size calculations for comparative studies of medical tests for detecting presence of disease. *Stat Med*. 2002;21(6):835-852. doi:10.1002/sim.1058

14. Pepe MS. Comparing disease screening tests when true disease status is ascertained only for screen positives. *Biostatistics*. 2001;2(3):249-260. doi:10.1093/biostatistics/2.3.249
