# Supplementary material for: Biomarker vs MRI-Enhanced Strategies for Prostate Cancer Screening: The STHLM3-MRI Randomized Clinical Trial
Source: JAMA Netw Open. 2024 Apr 22;7(4):e247131. doi: 10.1001/jamanetworkopen.2024.7131 (PMC11036143; doi:10.1001/jamanetworkopen.2024.7131)

Supplemental Online Content

Björnebo L, Discacciati A, Falagario U, et al. Biomarker-based vs MRI-enhanced strategies for prostate cancer screening: the STHLM3-MRI randomized clinical trial. *JAMA Netw Open.* 2024;7(4):e24713. doi:10.1001/jamanetworkopen.2024.7131

eTable 1. Relative Proportions of Men with MRI Scans, Biopsies, and Prostate Cancers According to Screening Strategies (Ignoring Biopsy Outcomes for Men in the MRI-Enhanced Group With Negative MRI Results who Underwent Biopsy due to a Stockholm3 Risk Score of 0.25 or Higher)

eTable 2. Relative Proportion of Men with MRI Scans, Biopsies, and Prostate Cancers According to Screening Strategies (Only Including Men Who Underwent Systematic Biopsies in the MRI-Enhanced Group)

eTable 3. Gleason Score at Biopsy vs at Prostatectomy in Biomarker-Based Group and MRI-Enhanced Group

eTable 4. Gleason Score at Systematic Biopsy vs Combined Systematic and Targeted Biopsy for Men in MRI-Enhanced Group who Underwent Both Types of Biopsies

eFigure. Gleason Score on Systematic Biopsy vs Combined Systematic and Targeted Biopsy for Men in MRI-Enhanced Arm who Underwent Both Types of Biopsies

This supplemental material has been provided by the authors to give readers additional information about their work.

eTable 1. Relative Proportions of Men with MRI Scans, Biopsies, and Prostate Cancers According to Screening Strategies (Ignoring Biopsy Outcomes for Men in the MRI-Enhanced Group With Negative MRI Results who Underwent Biopsy due to a Stockholm3 Risk Score of 0.25 or Higher)

	MRI-enhanced arm (N = 7609)	Biomarker arm (N = 5134)					
Outcome/Strategy	PSA ≥3 ng/ml	Stockholm3 ≥15%	Relative proportion (95% CI)	Stockholm3 ≥11%	Relative proportion (95% CI)	PSA ≥3 ng/ml	Relative proportion (95% CI)
Participants with elevated test, n (%)	929 (12)	413 (8.0)	0.66 (0.59 to 0.74)	680 (13)	1.08 (0.99 to 1.19)	603 (12)	0.96 (0.87 to 1.06)
MRIs, n (%)	846 (11)	1 (<0.1)	-	1 (<0.1)	-	1 (<0.1)	-
Biopsy procedures, n (%)	304 (4.0)	326 (6.3)	1.43 (1.23 to 1.66)	512 (10.0)	2.25 (1.97 to 2.56)	438 (8.5)	1.92 (1.67 to 2.20)
Benign biopsy procedures, n (%)	82 (1.1)	146 (2.8)	2.06 (1.61 to 2.64)	282 (5.5)	3.98 (3.19 to 4.97)	259 (5.0)	3.66 (2.92 to 4.57)
Gleason 6 cancers, n (%)	36 (0.5)	61 (1.2)	2.21 (1.49 to 3.27)	85 (1.7)	3.07 (2.12 to 4.45)	73 (1.4)	2.64 (1.80 to 3.86)
Gleason ≥3+4 cancers, n (%)	186 (2.4)	119 (2.3)	0.92 (0.73 to 1.15)	145 (2.8)	1.12 (0.90 to 1.38)	106 (2.1)	0.82 (0.65 to 1.03)
Gleason ≥4+3 cancers, n (%)	67 (0.9)	42 (0.8)	0.93 (0.63 to 1.36)	45 (0.9)	1.00 (0.68 to 1.45)	43 (0.8)	0.95 (0.65 to 1.39)
Referred for biopsy, no biopsy performed, n (%)	28 (0.4)	87 (1.7)	4.61 (3.01 to 7.04)	168 (3.3)	8.89 (5.97 to 13.25)	165 (3.2)	8.73 (5.86 to 13.02)
Number of biopsy cores, Mean	0.6	0.8	0.14 (0.03 to 0.24)	1.2	0.57 (0.45 to 0.69)	1.0	0.40 (0.28 to 0.51)
Number of post-biopsy hospitalizations, n (%)	12 (0.2)	10 (0.2)	1.14 (0.50 to 2.60)	22 (0.4)	2.51 (1.26 to 4.97)	17 (0.3)	1.94 (0.94 to 3.99)

eTable 2. Relative Proportion of Men with MRI Scans, Biopsies, and Prostate Cancers According to Screening Strategies (Only Including Men Who Underwent Systematic Biopsies in the MRI-Enhanced Group)

	MRI-enhanced arm (N = 7609)	Biomarker arm (N = 5134)					
Outcome/Strategy	PSA ≥3 ng/ml	Stockholm3 ≥15%	Relative proportion (95% CI)	Stockholm3 ≥11%	Relative proportion (95% CI)	PSA ≥3 ng/ml	Relative proportion (95% CI)
Participants with elevated test, n (%)	929 (12)	413 (8.0)	0.66 (0.59 to 0.74)	680 (13)	1.08 (0.99 to 1.19)	603 (12)	0.96 (0.87 to 1.06)
MRIs, n (%)	846 (11)	1 (<0.1)	-	1 (<0.1)	-	1 (<0.1)	-
Biopsy procedures, n (%)	338 (4.4)	326 (6.3)	1.43 (1.23 to 1.66)	512 (10.0)	2.25 (1.97 to 2.56)	438 (8.5)	1.92 (1.67 to 2.20)
Benign biopsy procedures, n (%)	133 (1.7)	146 (2.8)	1.63 (1.29 to 2.05)	282 (5.5)	3.14 (2.56 to 3.85)	259 (5.0)	2.89 (2.35 to 3.55)
Gleason 6 cancers, n (%)	58 (0.8)	61 (1.2)	1.56 (1.09 to 2.23)	85 (1.7)	2.17 (1.56 to 3.03)	73 (1.4)	1.87 (1.32 to 2.63)
Gleason ≥3+4 cancers, n (%)	147 (1.9)	119 (2.3)	1.20 (0.94 to 1.52)	145 (2.8)	1.46 (1.17 to 1.83)	106 (2.1)	1.07 (0.83 to 1.37)
Gleason ≥4+3 cancers, n (%)	49 (0.6)	42 (0.8)	1.27 (0.84 to 1.92)	45 (0.9)	1.36 (0.91 to 2.04)	43 (0.8)	1.30 (0.86 to 1.96)
Referred for biopsy, no biopsy performed, n (%)	28 (0.4)	87 (1.7)	4.61 (3.01 to 7.04)	168 (3.3)	8.89 (5.97 to 13.25)	165 (3.2)	8.73 (5.86 to 13.02)
Number of biopsy cores, Mean	0.7	0.8	0.08 (-0.02 to 0.19)	1.2	0.52 (0.40 to 0.64)	1.0	0.34 (0.23 to 0.46)
Number of post-biopsy hospitalizations, n (%)	13 (0.2)	10 (0.2)	1.14 (0.50 to 2.60)	22 (0.4)	2.51 (1.26 to 4.97)	17 (0.3)	1.94 (0.94 to 3.99)

eTable 3. Gleason Score at Biopsy vs at Prostatectomy in Biomarker-Based Group and MRI-Enhanced Group

		Gleason Score at prostatectomy				
**Group**		Gleason Score 6	Gleason Score 3+4	Gleason Score 4+3	Gleason Score ≥4+4	Total
MRI-enhanced arm (PSA ≥3 ng/ml)	Gleason Score biopsy, n (%)					
	Gleason Score 6	2 (7.7%)	18 (69%)	4 (15%)	2 (7.7%)	26 (100%)
	Gleason Score 3+4	3 (4.9%)	40 (66%)	15 (25%)	3 (4.9%)	61 (100%)
	Gleason Score 4+3	0 (0%)	3 (23%)	10 (77%)	0 (0%)	13 (100%)
	Gleason Score ≥4+4	0 (0%)	1 (10%)	2 (20%)	7 (70%)	10 (100%)
	Unknown	0 (0%)	13 (87%)	2 (13%)	0 (0%)	15 (100%)
	Total, n (%)	5 (4.0%)	75 (60%)	33 (26%)	12 (9.6%)	125 (100%)
Biomarker arm (Stockholm3 ≥15%)	Gleason Score biopsy, n (%)					
	Gleason Score 6	0 (0%)	11 (79%)	2 (14%)	1 (7.1%)	14 (100%)
	Gleason Score 3+4	0 (0%)	27 (87%)	4 (13%)	0 (0%)	31 (100%)
	Gleason Score 4+3	0 (0%)	1 (11%)	8 (89%)	0 (0%)	9 (100%)
	Gleason Score ≥4+4	0 (0%)	0 (0%)	3 (75%)	1 (25%)	4 (100%)
	Total, n (%)	0 (0%)	39 (67%)	17 (29%)	2 (3.4%)	58 (100%)
Biomarker arm (Stockholm3 ≥11%)	Gleason Score biopsy, n (%)					
	Gleason Score 6	0 (0%)	13 (81%)	2 (13%)	1 (6.3%)	16 (100%)
	Gleason Score 3+4	0 (0%)	34 (85%)	6 (15%)	0 (0%)	40 (100%)
	Gleason Score 4+3	0 (0%)	1 (10%)	8 (80%)	1 (10%)	10 (100%)
	Gleason Score ≥4+4	0 (0%)	0 (0%)	3 (75%)	1 (25%)	4 (100%)

		Gleason Score at prostatectomy				
**Group**		Gleason Score 6	Gleason Score 3+4	Gleason Score 4+3	Gleason Score ≥4+4	Total
	Total, n (%)	0 (0%)	48 (69%)	19 (27%)	3 (4.3%)	70 (100%)
Biomarker arm (Stockholm3 ≥15%)	Gleason Score biopsy, n (%)					
	Gleason Score 6	0 (0%)	11 (79%)	2 (14%)	1 (7.1%)	14 (100%)
	Gleason Score 3+4	0 (0%)	27 (87%)	4 (13%)	0 (0%)	31 (100%)
	Gleason Score 4+3	0 (0%)	1 (11%)	8 (89%)	0 (0%)	9 (100%)
	Gleason Score ≥4+4	0 (0%)	0 (0%)	3 (75%)	1 (25%)	4 (100%)
	Total, n (%)	0 (0%)	39 (67%)	17 (29%)	2 (3.4%)	58 (100%)
Biomarker arm (PSA ≥3 ng/ml)	Gleason Score biopsy, n (%)					
	Gleason Score 6	0 (0%)	10 (77%)	2 (15%)	1 (7.7%)	13 (100%)
	Gleason Score 3+4	0 (0%)	26 (87%)	4 (13%)	0 (0%)	30 (100%)
	Gleason Score 4+3	0 (0%)	2 (18%)	8 (73%)	1 (9.1%)	11 (100%)
	Gleason Score ≥4+4	0 (0%)	0 (0%)	3 (75%)	1 (25%)	4 (100%)
	Total, n (%)	0 (0%)	38 (66%)	17 (29%)	3 (5.2%)	58 (100%)

eTable 4. Gleason Score at Systematic Biopsy vs Combined Systematic and Targeted Biopsy for Men in MRI-Enhanced Group who Underwent Both Types of Biopsies

	Gleason Score at CBx					
	Benign biopsy	Gleason Score 6	Gleason Score 3+4	Gleason Score 4+3	Gleason Score ≥4+4	Total
Gleason Score at SBx, n (%)						
Benign biopsy	77 (73%)	7 (6.7%)	17 (16%)	3 (2.9%)	1 (1.0%)	105 (100%)
Gleason Score 6	0 (0%)	28 (54%)	20 (38%)	3 (5.8%)	1 (1.9%)	52 (100%)
Gleason Score 3+4	0 (0%)	0 (0%)	77 (89%)	7 (8.0%)	3 (3.4%)	87 (100%)
Gleason Score 4+3	0 (0%)	0 (0%)	0 (0%)	16 (80%)	4 (20%)	20 (100%)
Gleason Score ≥4+4	0 (0%)	0 (0%)	0 (0%)	0 (0%)	25 (100%)	25 (100%)
Total, n (%)	77 (27%)	35 (12%)	114 (39%)	29 (10%)	34 (12%)	289 (100%)

eFigure. Gleason Score on Systematic Biopsy vs Combined Systematic and Targeted Biopsy for Men in MRI-Enhanced Group who Underwent Both Types of Biopsies

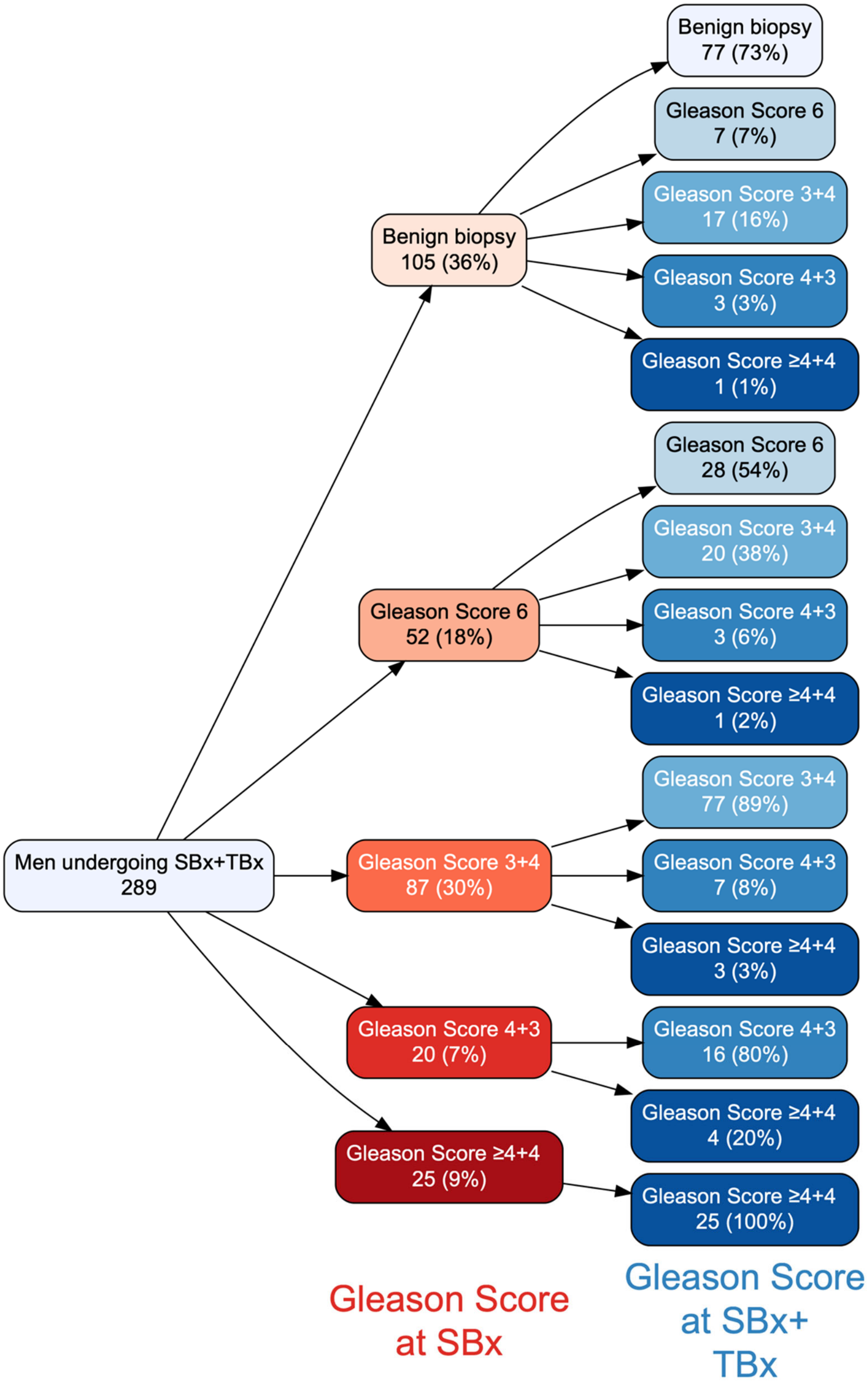

Supplement: Supplement 2. — eTable 1. Relative Proportions of Men with MRI Scans, Biopsies, and Prostate Cancers According to Screening Strategies (Ignoring Biopsy Outcomes for Men in the MRI-Enhanced Group With Negative MRI Results who Underwent Biopsy due to a Stockholm3 Risk Score of 0.25 or Higher) eTable 2. Relative Proportion of Men with MRI Scans, Biopsies, and Prostate Cancers According to Screening Strategies (Only Including Men Who Underwent Systematic Biopsies in the MRI-Enhanced Group) eTable 3. Gleason Score at Biopsy vs at Prostatectomy in Biomarker-Based Group and MRI-Enhanced Group eTable 4. Gleason Score at Systematic Biopsy vs Combined Systematic and Targeted Biopsy for Men in MRI-Enhanced Group who Underwent Both Types of Biopsies eFigure. Gleason Score on Systematic Biopsy vs Combined Systematic and Targeted Biopsy for Men in MRI-Enhanced Group who Underwent Both Types of Biopsies [file jamanetwopen-e247131-s002.pdf]
