# Supplementary material for: Biomarker vs MRI-Enhanced Strategies for Prostate Cancer Screening: The STHLM3-MRI Randomized Clinical Trial
Source: JAMA Netw Open. 2024 Apr 22;7(4):e247131. doi: 10.1001/jamanetworkopen.2024.7131 (PMC11036143; doi:10.1001/jamanetworkopen.2024.7131)
Supplement: Supplement 3. — Data Sharing Statement [file jamanetwopen-e247131-s003.pdf]

Data Sharing Statement

Björnebo. Biomarker vs MRI-Enhanced Strategies for Prostate Cancer Screening. *JAMA Netw Open*. Published April 22, 2024. doi:10.1001/jamanetworkopen.2024.7131

Data
Data available: Yes
Data types: Other (please specify)
Additional Information: Individual participant data underlying the results reported in this Article (in the text, tables, figures, and appendices) will be available after deidentification, together with the study protocol, statistical analysis plan, and analytic code. Data will be made available to researchers who provide a methodologically robust proposal, in which the aims are relevant and clear. Proposals can be submitted up to 24 months after publication of the Article and should be directed to tobias.nordstrom@ki.se. Only proposals with ethical approval will be granted access to the data.
How to access data: Please contact Tobias.Nordstrom@ki.se with requests.
When available: With publication

Supporting Documents
Document types: Other (please specify)
Additional Information: Please see above.
How to access documents: By request to Tobias.Nordstrom@ki.se
When available: With publication

Additional Information
Who can access the data: See above.
Types of analyses: See above.
Mechanisms of data availability: See above.
